# Reconstructing Spatiotemporal Trajectories of Visual Object Memories in the Human Brain

**DOI:** 10.1523/ENEURO.0091-24.2024

**Published:** 2024-09-26

**Authors:** Julia Lifanov-Carr, Benjamin J. Griffiths, Juan Linde-Domingo, Catarina S. Ferreira, Martin Wilson, Stephen D. Mayhew, Ian Charest, Maria Wimber

**Affiliations:** ^1^School of Psychology and Centre for Human Brain Health (CHBH), University of Birmingham, Birmingham B15 2TT, United Kingdom; ^2^Department of Experimental Psychology, Mind, Brain and Behavior Research Center (CIMCYC), University of Granada, 18011 Granada, Spain; ^3^Center for Adaptive Rationality, Max Planck Institute for Human Development, 14195 Berlin, Germany; ^4^Institute of Health and Neurodevelopment (IHN), School of Psychology, Aston University, Birmingham B4 7ET, United Kingdom; ^5^Département de Psychologie, Université de Montréal, Montréal, Quebec H2V 2S9, Canada; ^6^School of Psychology & Neuroscience and Centre for Cognitive Neuroimaging (CCNi), University of Glasgow, Glasgow G12 8QB, United Kingdom

**Keywords:** EEG, feature reconstruction, fMRI, memory retrieval, multivariate fusion

## Abstract

How the human brain reconstructs, step-by-step, the core elements of past experiences is still unclear. Here, we map the spatiotemporal trajectories along which visual object memories are reconstructed during associative recall. Specifically, we inquire whether retrieval reinstates feature representations in a copy-like but reversed direction with respect to the initial perceptual experience, or alternatively, this reconstruction involves format transformations and regions beyond initial perception. Participants from two cohorts studied new associations between verbs and randomly paired object images, and subsequently recalled the objects when presented with the corresponding verb cue. We first analyze multivariate fMRI patterns to map where in the brain high- and low-level object features can be decoded during perception and retrieval, showing that retrieval is dominated by conceptual features, represented in comparatively late visual and parietal areas. A separately acquired EEG dataset is then used to track the temporal evolution of the reactivated patterns using similarity-based EEG–fMRI fusion. This fusion suggests that memory reconstruction proceeds from anterior frontotemporal to posterior occipital and parietal regions, in line with a conceptual-to-perceptual gradient but only partly following the same trajectories as during perception. Specifically, a linear regression statistically confirms that the sequential activation of ventral visual stream regions is reversed between image perception and retrieval. The fusion analysis also suggests an information relay to frontoparietal areas late during retrieval. Together, the results shed light onto the temporal dynamics of memory recall and the transformations that the information undergoes between the initial experience and its later reconstruction from memory.

## Significance Statement

We combined EEG and fMRI to investigate which features of a visual object are reactivated when recalled from episodic memory and how the memory reconstruction stream unfolds over time and across the brain. Our findings suggest that relative to perception, memory retrieval follows a backward information trajectory along a conceptual-to-perceptual gradient and additionally relays retrieved information to multimodal frontoparietal brain regions. These findings address the fundamental question of whether memories are more or less truthful reconstructions of past events or instead are subject to systematic biases that prioritize some types of features over others. Our data suggest that episodic memory retrieval is a dynamic and highly reconstructive process with clear prioritization of abstract–conceptual over detailed–perceptual information.

## Introduction

Relative to visual perception, little is known about how the human brain reconstructs content that is retrieved from memory. We here investigated which features are preferentially reactivated where in the brain and when in time when visual objects are recalled from episodic memory.

Object perception triggers an information processing cascade along the ventral visual stream that follows a gradient of increasing abstraction: early processing stages are dominated by low-level perceptual representations, while later stages increasingly code for conceptual features (Kolb et al., 1995; Carlson et al., 2013; Cichy et al., 2014; Clarke and Tyler, 2015; Martin et al., 2018). After ∼300 ms, information reaches the hippocampus, where concept cells represent objects as highly abstract, perceptually invariant concepts (Quiroga, 2012). The hippocampus presumably binds together information from different sources (including dorsal stream) and serves the longer-term storage and retrieval of associative information (Eichenbaum, 2001; Rolls, 2010; Danker et al., 2017).

Computational models assume that the hippocampus then uses partial reminders to recover the missing, associatively linked information, a process termed pattern completion (Marr, 1971; Teyler and DiScenna, 1986; O’Reilly and McClelland, 1994; Rolls, 2010, 2013). Pattern completion in turn dictates that neocortical brain regions reinstantiate the various features of the relevant event (Marr, 1971; Teyler and DiScenna, 1986; Teyler and Rudy, 2007; Rolls, 2013; Moscovitch, 2008), presumably leading to the subjective reexperiencing of a past event (Tulving et al., 1983).

These early computational models describe memory reinstatement as a noisy but in essence veridical process, where neocortical information is reestablished the way it was represented during initial perception. Evidence for copy-like encoding-retrieval-similarities (ERS) (Staresina et al., 2012) comes from neuroimaging studies using similarity- or classification-based multivariate analyses (Danker and Anderson, 2010; Rissman and Wagner, 2012; Staresina et al., 2012), showing reinstatement of individual episodes (Ferreira et al., 2019), scenes (Wing et al., 2014), semantic category (Polyn et al., 2005), sensory modality (Griffiths et al., 2019), or task context (Jiang et al., 2020). While these findings support the central premise that hippocampal pattern completion is associated with a more or less truthful reinstatement of encoding patterns, recent studies emphasize that information is not necessarily decodable from the same regions during encoding and retrieval (Favila et al., 2020; Xue, 2022). A shift in the location of content decoding from sensory to multimodal frontoparietal areas suggests a representational transformation between perception and memory recall, respectively (Xiao et al., 2017; Favila et al., 2018, 2020; Xue, 2022). This memory transformation may go along with a semanticization of the retrieved content (Favila et al., 2020), in which conceptual features become prioritized over low-level perceptual ones (Ferreira et al., 2019; Lifanov et al., 2021).

Due to the divergent previous findings, our first goal in the present study was to decompose visual object memories into perceptual and conceptual components and to test if both types of features are equally reinstated, in the same areas representing these features during encoding. Moving beyond spatial localization, we asked next how the neocortical reinstatement process unfolds over time, and whether different features of a memory are reactivated with different temporal priority. Such prioritization could naturally emerge from the hippocampus’ differential connectivity with neocortical regions during retrieval. Once pattern completion is initiated, most information is relayed via the subiculum to the entorhinal cortex which in turn projects back to other cortical areas (Chrobak et al., 2000; Rolls, 2013; Staresina and Wimber, 2019). A majority of direct hippocampal and entorhinal efferents terminate in late areas of the visual processing hierarchy where representations cluster in abstract–semantic categories, while there are few connections to low-level sensory areas (Insausti and Muñoz, 2001; Dalton et al., 2022). We therefore expect that memories are initially reconstructed on a conceptual level, and that information coded in lower-level visual areas can only be reached at later stages of memory reconstruction. Indeed recent studies using EEG decoding and behavioral responses demonstrated a temporal prioritization of conceptual over perceptual features during memory retrieval (Linde-Domingo et al., 2019; Lifanov et al., 2021), mirroring the hierarchy during perception of the same stimuli, similarly to mental imagery (Horikawa and Kamitani, 2017; Dijkstra et al., 2020). Whether the observed conceptual-to-perceptual gradient of feature recall truly maps onto a sensory backpropagation stream remains an open question.

The present study combined fMRI with EEG pattern analysis to test if recalling an object from memory elicits a backward processing cascade relative to perception. An associative recall paradigm with visual object stimuli (Linde-Domingo et al., 2019, Lifanov et al., 2021) allowed us to differentiate between perceptual and conceptual object features. First, based on fMRI patterns only, we compared where in the brain these low- and high-level feature dimensions can be decoded during retrieval as opposed to encoding. Second, using EEG–fMRI fusion to resolve spatial activation patterns in time, we asked whether the object information reconstructed during retrieval propagates backward (i.e., anterior to posterior) along the ventral visual stream, from high-level conceptual to low-level sensory areas.

## Materials and Methods

### Participants

We acquired fMRI data of 37 right-handed, fluent English-speaking participants at the University of Birmingham (26 females, 11 males, mean age (*M*_age_) = 23.33, standard deviation (SD_age_) = 3.89; one participant did not indicate their age). The a priori planned sample size for the full EEG–fMRI dataset was *n *= 24 subjects (as in Linde-Domingo et al., 2019). However, due to poor data quality and technical failures in 13 of the simultaneous EEG datasets, additional subjects were recorded leading to a larger sample size for the fMRI data alone. Three subjects were excluded from the fMRI analysis due to failed scanning sequences, and three additional subjects were excluded due to extensive motion within the scanner, exceeding the functional voxel size, such that 31 fMRI datasets remained for analysis. All participants were informed about the experimental procedure, underwent imaging safety screening, and signed an informed consent. The research (ethics code ERN_11-0429AP70) was approved by the STEM ethics committee of the University of Birmingham.

Due to poor data quality in the simultaneously acquired EEG set (see below, Analyses that did not work), we excluded the entire simultaneous EEG data from the analysis and instead used a previously published EEG dataset including the same stimulus set and a nearly identical associative learning and retrieval paradigm (Linde-Domingo et al., 2019). This additional dataset included 24 further participants with a clean, out-of-scanner EEG. For further information on the EEG data sample and the related ethical procedures, we refer to the previous work (Linde-Domingo et al., 2019).

### Material

**Figure 1.**
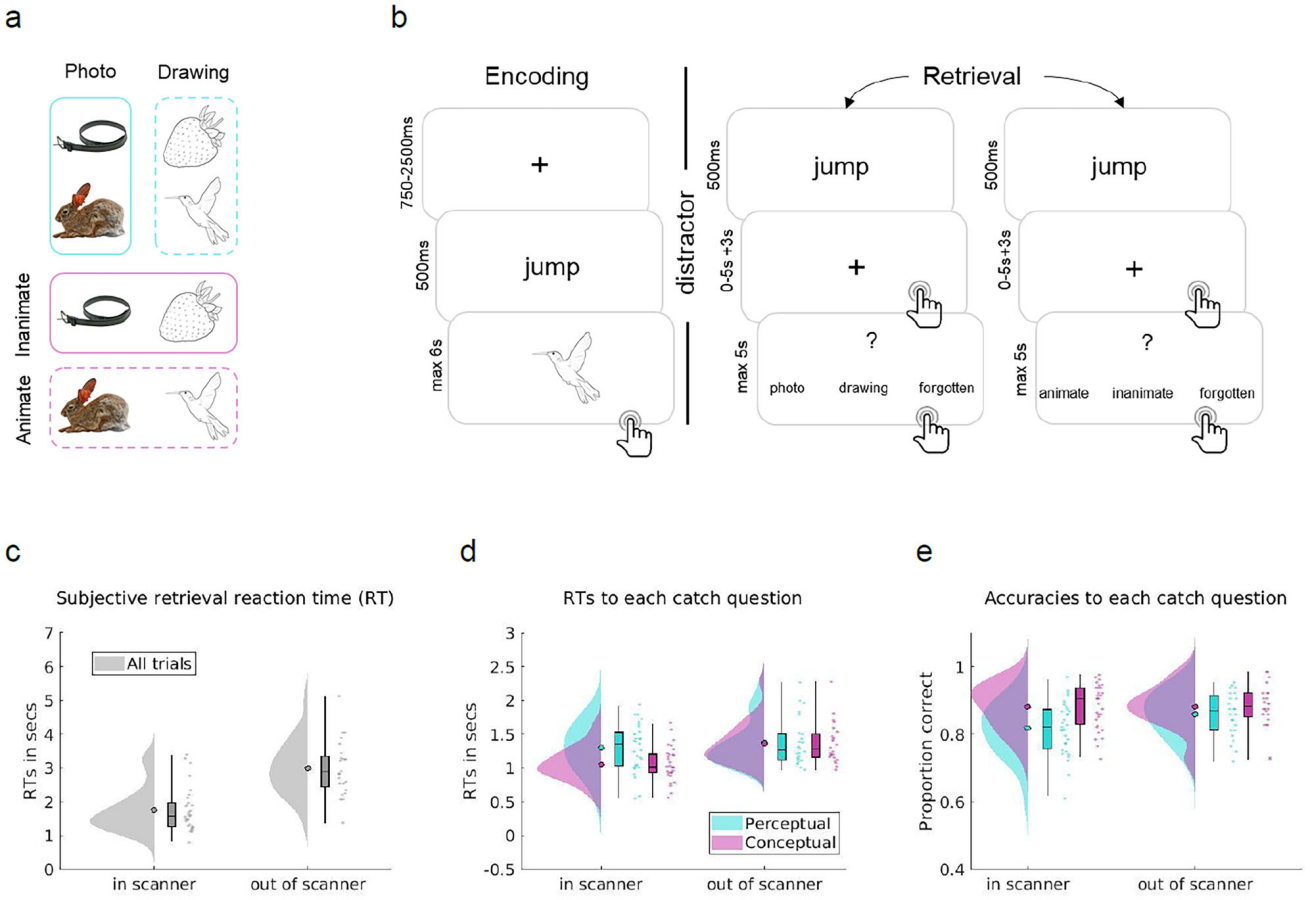
Overview of stimuli and task. ***a***, Design of the stimuli. The 128 pictures used in any given participant were orthogonally split into 64 drawings and 64 photographs, out of which 32 were animate and 32 inanimate objects, respectively. Each object could thus be classified along a perceptual (photo/drawing, cyan) or conceptual (animate/inanimate, magenta) dimension. ***b***, One prototypical task block of the paradigm. At encoding, participants were asked to associate verb–object pairings and indicate the successful formation of an association by button press. After a 20 s distractor task, participants did a memory test where on each trial, they recalled the previously associated object upon presentation of a verb cue and indicated successful subjective recollection by button press (referred to as retrieval button press). Then participants were asked to hold the mental image of the object in mind for a further 3 s, before answering a perceptual or conceptual catch question. Each association was tested twice during retrieval, once with a perceptual, and once with a conceptual question, with several intervening test trials in between repetitions of the same association. Participants performed 16 task blocks overall, with eight novel associations per block. Stimuli illustrated are chosen from the BOSS database (Brodeur et al., 2010, 2014; https://creativecommons.org/licenses/by-sa/3.0/, Photos: Copyright © 2014 Brodeur et al.) and customized with free and open-source GIMP software (The GIMP Development Team, 2019; https://www.gimp.org; Linde-Domingo et al., 2019). Figure adapted from Lifanov et al. (2021) and Linde-Domingo et al. (2019). ***c–e***, Behavioral data acquired within (EEG–fMRI group) and out of scanner (EEG only group). ***c***, Subjective retrieval RTs, ***d***, RTs for each type of catch question. ***e***, Catch question accuracies for the two question types. Filled circles represent the overall *M*; boxplots represent median and 25th and 75th percentiles; whiskers represent 2nd and 98th percentile; dots represent the *M* of individual subjects. Gray represents all trials; cyan represents responses to perceptual and magenta to conceptual questions. In-scanner data come from *n* = 31 independent subjects, out-of-scanner data from *n* = 24 independent subjects. Figures made using RainCloud plots Version 1.1 (https://github.com/RainCloudPlots/RainCloudPlots, M. Allen et al., 2018, 2021).

The paradigm was a visual verb–object association task (Linde-Domingo et al., 2019) adapted for fMRI in terms of timing. Stimuli included 128 action verbs and 128 pictures of everyday objects (Linde-Domingo et al., 2019). Action verbs were chosen because they do not elicit concrete object images in themselves but are still easy to associate with the object images. Importantly, all object images existed in two perceptual categories, a colored photograph from the BOSS database (Brodeur et al., 2010, 2014) or a black line drawing version of a respective photograph created by Linde-Domingo et al. (2019) by means of the free and open-source GNU Image Manipulation Program (GIMP) software (The GIMP Development Team, 2019). Furthermore, each object belonged to one of the two conceptual categories, i.e., animate versus inanimate. We selected 128 images per participant according to a fully balanced scheme, such that each combination of perceptual and conceptual categories included the same number of pictures (32 animate photographs, 32 animate drawings, 32 inanimate photographs, 32 inanimate drawings; Fig. 1*a*). With respect to the later fusion with the out-of-scanner EEG data, it is important to note that while both experiments used the same set of 128 objects, the same object could appear in different perceptual versions between participants, due to the pseudorandomized image selection. For example, an image of a camel could be shown as a photograph to one participant and as a line drawing to another. Action verbs were randomly assigned to images in each participant and were presented together with pictures centrally overlaid on a white background.

### Procedure

Before the session, participants were informed about the experimental procedure and asked to perform a training task block in front of the computer. After completion of the training, the experimental session in the fMRI scanner included four runs with four task blocks each, summing up to a total of 16 task blocks. A typical block in the training and experimental task included the encoding of eight novel associations, a 20 s distractor task, and 16 retrieval trials (two repetitions per association). A 3 min break was included after each fMRI run, in which participants were asked to rest and close their eyes. In total, it took participants ∼70 min to perform the entire task.

#### Encoding

In the encoding phases (Fig. 1*b*), participants were instructed to study eight novel verb–object pairings in a random order. A trial started with a fixation cross presented for a jittered period between 750 and 2,500 ms. The action verb was presented for 500 ms before an object was shown for a maximum duration of 6 s. To facilitate learning, participants were instructed to form a vivid visual mental image using the verb–object pairing and, once formed, to press a button with their thumb, which moved the presentation on to the next trial. Importantly, due to the artificial setting where there is no predictive structure to the events, we expected very limited top–down feedback and hence a dominating feedforward sweep during perception early on.

#### Distractor

After each encoding phase, participants performed a self-paced distractor task for 20 s, indicating as fast as possible whether each of the consecutively presented numbers on the screen was odd or even, pressing a button with their index or middle finger, respectively. Feedback on the percentage of correct responses was provided at the end of each distractor phase.

#### Retrieval

In the retrieval phases, participants were instructed to recall the previously associated objects upon presentation of the corresponding verb (Fig. 1*b*). Trials started with the presentation of a fixation cross, jittered between 500 and 1,500 ms and followed by a previously encoded action verb, presented for 500 ms. Cued with the verb, participants were instructed to recall the paired object within a maximum of 5 s, while a black fixation cross was presented on the screen. Note that this maximum duration was shorter than in the original task (Linde-Domingo et al., 2019), which might have caused participants to react faster and possibly explains reaction time (RT) differences between the in-scanner and out-of-scanner datasets (Fig. 1*c*). Participants were asked to indicate the time point of image recall by button press with their thumb, at which point the black fixation cross turned gray and was presented for an additional 3 s. This retrieval button press was meant to mark the time point of subjective recollection. During the remaining 3 s, participants were asked to hold the mental image of the object in mind as vividly as possible. Last, they were asked about the perceptual (was the object a photograph or a line drawing?) or conceptual (was the object animate or inanimate?) features of the recalled object, answering with their index or middle finger within a maximum response period of 5 s. During the presentation of the catch question, participants also had the option to indicate with their ring finger that they forgot the features of the corresponding object. Importantly, each encoded stimulus was retrieved twice, once with a perceptual and once with a conceptual question. Trial order was pseudorandom within the first and second set of repetitions, with a minimum of two intervening trials before a specific object was recalled for the second time. The order of catch questions was counterbalanced across repetitions such that half of the associations were first probed with a perceptual question, and the other half was first probed with a conceptual question.

### fMRI data acquisition

FMRI scanning was performed in a 3 Tesla Philips Achieva MRI scanner with a 32-channel SENSE head coil at the Birmingham University Imaging Centre (now the Centre for Human Brain Health). Slices covered the whole head. T1-weighted anatomical scans were collected with an MP-RAGE sequence [1 mm isotropic voxels; 256 × 256 matrix; 176 slices; no interslice gap; repetition time (TR), 7.5 ms; field of view (FOV), 256 × 256 × 176 mm; flip angle (FA), 7°; echo time (TE), 3.5 ms]. T2*-weighted functional images were acquired by a dual-echo EPI pulse sequence with a low specific absorption rate to optimize the image quality in regions highly susceptible for distortions by a long readout gradient (3 × 3 × 3.5 mm; 64 × 64 matrix; 34 slices; no interslice gap; TR, 2.2 s; full head coverage; FOV, 192 × 192 × 119 mm; FA, 80°; TE1, 12 ms; TE2, 34 ms; for dual-echo information, see also Halai et al., 2014, 2015; Kirilina et al., 2016). Slices were acquired in a continuous descending fashion. Furthermore, we collected 200 resting-state volumes, 50 after each of the four task runs, used for the later combining of dual-echo images with the short and long TEs. During the acquisition of the functional scans, the helium pump was switched off to prevent contamination of the EEG by the compressor artifact at ∼20–30 Hz. Stimulus timings were jittered in relation to the acquired scans. The task was presented to participants through a mirror system in the scanner and a JVC SX 21e projector with resolution 1,280 × 1,024 at 60 Hz (https://de.jvc.com/). Participants’ heads were extra padded to minimize movement artifacts. The stimulus presentation, and the collection of timing and accuracy information, was controlled by scripts written in MATLAB (The MathWorks Inc., 2016, https://www.mathworks.com) and Psychophysics Toolbox (Brainard, 1997; Pelli, 1997; Kleiner et al., 2007; version 3). Responses were logged by NATA response boxes (https://natatech.com/).

### Out-of-scanner EEG data acquisition

As previously stated, a simultaneous EEG dataset was acquired during the fMRI session, but this EEG dataset was too noisy to decode retrieval-related information. We therefore decided to instead use an out-of-scanner EEG dataset for data fusion. This out-of-scanner data, used for all analyses reported here, originated from a previous publication (Linde-Domingo et al., 2019). We give a short description of the EEG data acquisition in the following and of the EEG preprocessing further below. More details can be found in the original publication.

For EEG data collection, 128 electrodes of silver metal and silver chloride were used. Furthermore, an Active-Two amplifier system and the ActiView acquisition software aided data recording (BioSemi; https://www.biosemi.com/). Psychophysics Toolbox (Brainard, 1997; Pelli, 1997; Kleiner et al., 2007; version 3) and MATLAB (The MathWorks Inc., 2014, https://www.mathworks.com) were used for task presentation and response collection (Linde-Domingo et al., 2019).

### Analyses

#### Behavior

Behavioral data were analyzed by MATLAB (The MathWorks Inc., 2017, https://www.mathworks.com). For the inspection of RTs and accuracies, the behavioral data were preprocessed as follows. For the RT analysis, all trials with incorrect responses to the catch question were removed first. Additionally, catch question RTs of correct trials were removed if they were faster than 200 ms or exceeding the average RT of a participant in a given set of repetitions (first or second cycle) by more than three times the SD (Linde-Domingo et al., 2019; Lifanov et al., 2021). For the analysis of the accuracy data, trials faster than 200 ms and objects with a missing response for either of the two questions were excluded in the corresponding cycle (Lifanov et al., 2021). The same procedures were applied to the data of the out-of-scanner EEG participants, who performed a nearly identical task. However, note that out-of-scanner EEG participants only went through one cycle of retrieval.

#### FMRI data preprocessing

We used MATLAB (The MathWorks Inc., 2017, https://www.mathworks.com) and SPM 12 (Penny et al., 2007) for the preprocessing and the analysis of fMRI data. All functional images were first realigned based on three motion and three rotation parameters, unwarped, and slice time corrected to the middle slice in time.

After these initial preprocessing steps, images obtained during the task at two echo times were combined as a weighted average (Poser et al., 2006). Importantly, the relative weights were obtained from the SNR of 200 resting-state volumes per echo and its corresponding TE as follows:

$$w(\mathrm{CNR}{)}_{n}=\frac{\mathrm{SN}R_{n}\;*\;TE_{n}}{\sum_{n}\;\mathrm{SN}R_{n}\;*\;TE_{n}},$$

where *w* is the weight for an individual voxel, $\mathrm{SN}R_{n}$ is the signal-to-noise ratio here calculated as ratio of *M* to SD of the given voxel calculated over time at the *n*th echo, and TE is the readout time of the *n*th echo.

Weights obtained from the resting state were then used to combine task volumes from both echoes as follows:

$$S=w_{1}\;*\;S_{1}+w_{2}\;*\;S_{2},$$

where *S* is the final signal of an individual voxel over time, calculated by summing the weighted signals of both echoes. These methods and equations were applied as described in work on BOLD contrast optimization by multiecho sequences (Poser et al., 2006). Combined image structures were written to NIFTI files (https://nifti.nimh.nih.gov/) by Tools for NIfTI and ANALYZE image (Shen, 2014, https://www.mathworks.com/matlabcentral/fileexchange/8797-tools-for-nifti-and-analyze-image).

Anatomical images were segmented, coregistered with the functional images, and normalized into a standard MNI template in SPM 12 (Penny et al., 2007). Then, after the combination of functional images, the T2* images were also normalized into MNI space, using the T1-based normalization parameters. Finally, EPI images were smoothed for the univariate GLM analysis with a Gaussian spatial filter of 8 mm full-width at half-maximum (FWHM). Note that multivariate analyses were performed on unsmoothed data in native space (Weaverdyck et al., 2020).

##### ROIs

Regions of interest (ROIs) were created from templates in MNI space as available in the WFU PickAtlas v3.0 (Lancaster et al., 1997, 2000; Tzourio-Mazoyer et al., 2002; Maldjian et al., 2003, 2004). These ROIs were then fitted to individual brains by applying the reversed T1-based normalization parameters (see above, FMRI data preprocessing) to the ROI masks (as in Chang and Glover, 2010). To define the anatomical masks, we used Brodmann areas (BAs, Brodmann, 1909) from the Talairach Daemon database atlases (Lancaster et al., 1997, 2000) and automated anatomical labeling (AAL, Tzourio-Mazoyer et al., 2002). The anatomical masks used for our analyses included an early visual mask (V1/V2), consisting of BAs 17 and 18; a human inferior temporal (hIT) mask, consisting of BAs 19 and 37; a temporal pole mask, consisting of superior and middle temporal pole regions as defined by AAL; a medial temporal lobe (MTL) mask, consisting of BAs 28, 34, 35, and 36 and AAL rhinal sulcus and parahippocampal gyrus; a superior parietal lobe mask, consisting of BA 7; a lateral (inferior) parietal lobe mask, consisting of BAs 39 and 40; and a medial parietal lobe mask, consisting of BAs 29 and 30.

#### EEG data preprocessing

EEG data (Linde-Domingo et al., 2019; see section “Data availability”) were preprocessed in the MATLAB (The MathWorks Inc., 2014, https://www.mathworks.com) and the FieldTrip toolbox (Oostenveld et al., 2010; Donders Institute for Brain, Cognition and Behaviour, Radboud University Nijmegen, the Netherlands. See http://www.ru.nl/neuroimaging/fieldtrip, version from 3 Aug. 2017). During epoching, different temporal references were used for encoding and retrieval. Encoding epochs were stimulus locked to the onset of the object image, while retrieval timelines were locked to the subjective retrieval button press, in order to observe the reactivation stream leading up to the subjective experience of recollection. These epochs were created with a length of 2 s (−500 before to 1,500 ms after object onset) for encoding and 4.5 s (−4 s before to 500 ms after retrieval button press) for retrieval. Line noise was removed by a finite impulse response filter with a band-stop between 48 and 52 Hz. A high-pass filter with a cutoff frequency of 0.1 Hz was applied to remove slow temporal drifts and a low-pass filter with a cutoff frequency of 100 Hz to remove high-frequency noise. Individual artifactual trials and bad electrodes were rejected manually. Remaining artifacts were removed by independent component analysis, after which any excluded electrodes were reintroduced by interpolation. The referencing of the data was set to the average across all scalp channels.

After this step, we implemented additional preprocessing steps using MATLAB (The MathWorks Inc., 2017, https://www.mathworks.com) and the FieldTrip toolbox (Oostenveld et al., 2010; Donders Institute for Brain, Cognition and Behaviour, Radboud University Nijmegen, the Netherlands. See http://www.ru.nl/neuroimaging/fieldtrip, version from 16 November 2017) to prepare data for the specific fusion analyses of this paper. The encoding data were baseline corrected by subtracting the average signal in the prestimulus period (−0.2 to −0.1 s) for all poststimulus time points, separately per electrode. The retrieval data were baseline corrected by whole-trial demeaning, since retrieval trials had no obvious, uncontaminated baseline period. The EEG time series data were then downsampled to 128 Hz and temporally smoothed with a moving average with a time window of 40 ms.

#### fMRI univariate analyses

We first performed a univariate GLM analysis and subsequent *t*-contrasts on the fMRI data to reveal activation differences between the two perceptual and the two conceptual categories (Extended Data Fig. 2-2, Extended Data Tables 2-7–2-9). The GLM included the four main regressors (drawing-animate, photograph-animate, drawing-inanimate, and photograph-inanimate), with separate regressors for encoding, first retrieval, and second retrieval, using all trials. We used stick functions locked to the object onset to model encoding trials and boxcar functions with a duration of 2.5 s locked to the cue onset to model retrieval trials. We added one regressor each for the presentation of verbs, button presses, and perceptual and conceptual catch questions as well as nuisance regressors for head motion, scanner drift, and run means. After computing the GLM for each subject, a sample-level ANOVA was computed with the perceptual (photograph vs line drawing) and conceptual (animate vs inanimate) dimensions as within-subject factors. Planned comparisons contrasting photographs versus drawings and animate versus inanimate objects were carried out in subsequent *t*-contrasts separately at encoding and retrieval for all trials. The *t*-contrasts for retrieval trials were performed using both the first and second retrieval trials together.

#### fMRI multivariate analyses

As preparation for the multivariate analyses, we performed a GLM, modeling individual object-specific regressors for encoding, and, for each of the two retrieval repetitions separately, adding regressors of no interest for the presentation of verbs, button presses, and catch question onsets, as well as nuisance regressors for head motion, scanner drift, and run means (one regressor per variable containing all onsets; Griffiths et al., 2019). We used stick functions locked to the object onset to model encoding trials and boxcar functions with a duration of 2.5 s locked to the cue onset to model retrieval trials. The resulting beta weights were transformed into *t* values for all subsequent multivariate analyses (Misaki et al., 2010).

##### fMRI searchlight LDA

To investigate where in the brain activity patterns differentiated between the two perceptual and the two conceptual categories, we performed a volumetric linear discriminant analysis (LDA) searchlight analysis on the non-normalized and unsmoothed fMRI data of each participant individually using the searchlight function of the representational similarity analysis toolbox (RSA, Nili et al., 2014; https://www.mrc-cbu.cam.ac.uk/methods-and-resources/toolboxes/). The LDA was calculated using the MVPA-Light toolbox (Treder, 2020; https://github.com/treder/MVPA-Light). This was done at each center voxel of a sphere, where object-specific *t* values of the voxels within a 3D searchlight radius of 12 mm were used as feature vectors. Using these feature vectors, we classified perceptual (photo vs drawing) and conceptual (animate vs inanimate) categories by a fivefold LDA with five repetitions, preserving class proportions, separately for encoding and retrieval using all trials. Individual accuracy maps were normalized to MNI space and spatially smoothed with a 10 mm FWHM Gaussian kernel, before second-level *t* tests were performed to statistically compare voxel-specific classification accuracies against 50% chance performance. Finally, the results were plotted on an MNI surface template brain.

##### fMRI correlation-based RSA

As preparation for the fusion of EEG and fMRI data, we performed an RSA (Kriegeskorte et al., 2006, 2008; Kriegeskorte, 2009; Kriegeskorte and Kievit, 2013; Nili et al., 2014; https://www.mrc-cbu.cam.ac.uk/methods-and-resources/toolboxes/) on the non-normalized and unsmoothed fMRI data. FMRI-based *t*-maps corresponding to unique objects were arranged in the same order for all participants. As mentioned earlier, object correspondence across participants was only given on the level of object identity (and thus also conceptual category), but not perceptual format. In other words, all participants saw an image of a camel (i.e., an animate object) at some point in the experiment, but the camel could be presented as a photograph to some participants and as a line drawing to others. For each voxel and its surrounding neighbors within a radius of 12 mm, we extracted object-specific *t* value patterns resulting from the appropriate GLM and arranged these as one-dimensional feature vectors. Using these feature vectors, we calculated the Pearson’s correlation distance (1 − *r*) between each pair of objects at each voxel location, separately for encoding and retrieval. The resulting representational dissimilarity matrix (RDM) maps were saved and used at a later stage for the searchlight fusion with the EEG data.

In a similar fashion to the searchlight approach, RDMs were also calculated for the predefined set of anatomical ROI in the non-normalized and unsmoothed individual functional datasets (see above, ROIs). The resulting ROI RDMs were used at a later stage for the ROI fusion.

#### EEG multivariate analyses

Our next step was to construct an RDM for each time point of the EEG recordings, resulting in one timeline representing the similarity structure of our object dataset across all subjects. Cells of the RDM represented the pairwise discriminability of object pairs, based on a cross-subject classification of object identity using the MVPA-Light toolbox (Treder, 2020; https://github.com/treder/MVPA-Light). To compute this matrix, we arranged object-specific trials in the same order between all participants, independent of their perceptual format, and up to 24 repetitions of each individual object across participants were used for the pairwise classification (note that after trial removal in the preprocessing, an average of *M* = 3.09 trials per object were missing in the encoding and *M* = 3.93 trials per object were missing in the retrieval data). Specifically, we performed a time-resolved LDA using as feature vectors the EEG amplitude values from the 128 electrodes, at a given time bin, and participants as repetitions of the same object. To tackle problems of overfitting, we used automatic shrinkage regularization as implemented in the MVPA-Light toolbox (Treder, 2020, https://github.com/treder/MVPA-Light). We used the LDA to classify object identity among each pair of objects at each time bin, again with a fivefold cross-validation, preserving class proportions. The resulting discrimination accuracies were entered in a single time-resolved RDM structure representing the dissimilarity between individual objects of our stimulus pool across participants over time. This classification procedure was performed independently for encoding and retrieval.

Before using the EEG-based RDMs for our data fusion, we also wanted to assess statistically how much information about object identity can be decoded from the EEG signals themselves. We therefore first calculated the average classification performance across all pairwise accuracies as a descriptive measure. We then tested if the pairwise accuracies resulting from the “real” classification with correct object labels were significantly larger than the pairwise accuracies that resulted from a classification with permuted object labels. This test was performed in two steps. First, we created 25 classification-based RDMs (same classification procedure as for the “real” RDM time course) but each one with randomly permuted object labels, keeping a given label permutation consistent across time in order to preserve the autocorrelation of the EEG time series. The 25 permutations were averaged to form a single “baseline” RDM time course.

As a second step, we used a cluster-based permutation test to find clusters with temporally extended above-chance decoding accuracy. This cluster-based permutation test compared the *t*-statistic of each time point resulting from a “real matrix” versus “baseline matrix” *t* test with the *t*-statistics computed from a “real matrix” versus “baseline matrix” *t* test, this time shuffling the “real” and “baseline” cells between the two matrices (consistent shuffling across time, independent samples *t* test to control for unequal variance, 1,000 permutations, cluster-definition threshold of *p* < 0.05, as used in previous publications; Cichy and Pantazis, 2017; Cichy et al., 2019; Dobs et al., 2019). Note that the variance in the *t* tests for each time bin comes from the pairwise accuracies contained in the cells of the two (“real” and “baseline”) classification matrices. All remaining analyses were performed using the (“real”) classification matrix with the correct object labels.

Next, we tested whether object discriminability systematically differs between objects coming from the same or different conceptual classes (i.e., animate and inanimate objects) within the classification with correct labels. We thus calculated the average accuracies of pairwise classes within and between conceptual categories as a descriptive measure. Using another cluster-based permutation test (paired-sample *t* test, 1,000 permutations, cluster-definition threshold of *p* < 0.05), we statistically tested for differences of within- against between-category accuracies over time. This analysis was conducted for conceptual classes only, as there was no correspondence of perceptual class between subjects. The encoding analyses focused on the time period from 0 to 1.5 s after the object onset, while the retrieval analyses focused on the time period from −3 to −1 s before retrieval button press. We based this latter time window of interest on previous findings (Linde-Domingo et al., 2019) which indicated perceptual and conceptual decoding peaks prior to −1 s before button press. All cluster permutation tests were implemented by means of the permutest toolbox (Gerber, 2019, https://www.mathworks.com/matlabcentral/fileexchange/71737-permutest; Maris and Oostenveld, 2007).

#### EEG–fMRI data fusion

##### ROI fusion

Two distinct approaches were used for the fusion analyses, with either the fMRI data or the EEG data serving as starting point. The first approach used the fMRI patterns from a given ROI as a starting point, and we thus refer to it as ROI fusion. One RDM was created per participant per ROI, representing the similarity structure (i.e., RDM) in a given anatomical brain region. This ROI RDM was then Spearman's-rank-correlated with the RDM from each time bin of the single, EEG-based RDM time course that represents the pooled similarity structure across subjects (see cross-subject classification described above). Correlations and classification accuracies were Fisher's *z*-transformed before the data fusion (as in Griffiths et al., 2019). This analysis resulted in one correlation time course per individual ROI per subject who took part in the fMRI experiment (*n *= 31). The EEG–fMRI correlations were only computed for those cells of the matrix that an individual participant from the fMRI study remembered correctly. To test for statistical significance, we then contrasted the 31 correlation time courses against zero, using a cluster-based permutation test with 1,000 permutations and a cluster-definition threshold of *p* < 0.05, correcting for multiple comparisons in time as in previous studies (Cichy and Pantazis, 2017; Cichy et al., 2019; Dobs et al., 2019).

To test for a sequential information progression over the ventral visual stream regions, we implemented a linear regression on the cumulative sums of the ROI time courses (Michelmann et al., 2019). This analysis complements the ROI fusion approach and was designed specifically to detect a cascade of serial processing stages, applied here to the representational similarity across a specific order of ROIs. Furthermore, it has been shown to be a strong method to identify such cascades within participants (Michelmann et al., 2019). To do so, we first calculated the cumulative sum of the ROI time courses over the time period from 0 to 1.5 s after the image onset for encoding and from −3 to −1 s before button press for retrieval. To compensate for total correlation differences between ROIs and make them more comparable, the cumulative sum of each ROI time course was normalized to an area under the curve that equals 1 (Michelmann et al., 2019). For each time point, a linear regression was fitted across the cumulative correlation values of the ROIs within subjects. The resulting slopes of all subjects were then tested against zero in a one-sided cluster–based permutation test (with 1,000 permutations and a cluster-definition threshold of *p* < 0.05). This method enabled us to test for a forward stream during encoding and a backward stream during retrieval (Fig. 4*e*,*f*). An example of the rationale is illustrated in Figure 3. In the case of a forward stream, the ROIs along the ventral visual stream activate sequentially from early toward late regions. Therefore, earlier regions (e.g., V1-hIT) show a higher cumulative sum than later regions (e.g., temporal pole—MTL) at 0.5 s after the stimulus onset (and other time points). A linear fit across ROIs at 0.5 s will therefore show a significant negative slope. According to this rationale, a backward stream across the same regions would result in a significant positive slope, while data peaks merely based on noise would reveal a slope near zero. Since we expected a forward stream at encoding and a backward stream at retrieval, we used one-sided tests to confirm if the slope differs from zero (<0 at encoding, >0 at retrieval). The sequential ordering of our ROIs was based on literature describing the hierarchical organization of the ventral visual stream in anatomical and functional neuroimaging studies (Felleman and Essen, 1991; Clarke et al., 2013; Cichy et al., 2014, 2016, 2017; Leonardelli and Fairhall, 2022).

##### Searchlight fusion

The second, complementary fusion approach offers higher spatial resolution and a whole-brain perspective on how encoding and retrieval patterns emerge over time. Here, we used the EEG-based RDMs from each time point as starting point and then searched for matching similarity structures across the entire brain, using a volumetric searchlight analysis on each individual's fMRI data (Kriegeskorte et al., 2006, 2008; Kriegeskorte, 2009; Kriegeskorte and Kievit, 2013; Nili et al., 2014, https://www.mrc-cbu.cam.ac.uk/methods-and-resources/toolboxes/). We refer to this method as a (time-resolved) searchlight fusion. A second-order correlation was computed between the (classification-based) EEG RDM from each time bin and the (correlation-based) fMRI RDMs for each center voxel and its neighbors within a searchlight radius of three voxels, separately for encoding and retrieval. This analysis results in a “fusion movie” (i.e., a time-resolved brain map) for each participant who took part in the fMRI experiment. The searchlight fusion for retrieval data was performed for correct trials within the fMRI data only (same as above). The fused data was Fisher's *z*-transformed. The searchlight fusion was performed using the Python Representational Similarity Analysis toolbox (RSA Group, 2019, https://rsatoolbox.readthedocs.io/en/latest/, https://github.com/rsagroup/rsatoolbox) written in Python (van Rossum, 1995), using the sys and os module, SciPy (Virtanen et al., 2020a,b), NumPy (Harris et al., 2020), and nibabel (https://nipy.org/nibabel/; https://github.com/nipy/nibabel/releases).

To test for significant EEG–fMRI pattern similarity at individual voxels, we normalized the correlation maps to MNI space, smoothed them with a 10 mm FWHM Gaussian kernel and then tested them in a one-sample *t* test against zero at each single time bin. The *t* test included a spatial maximal permuted statistic correction combined with a threshold-free cluster enhancement (Nichols and Holmes, 2002; Smith and Nichols, 2009). This cluster-based analysis was performed using the toolbox MatlabTFCE (Thornton et al., 2016, http://markallenthornton.com/blog/matlab-tfce/) with 1,000 permutations, a height exponent of 2, an extent exponent of 0.5, a connectivity parameter of 26, and a step number for cluster formation of 0.1 as suggested by Smith and Nichols (2009). The analysis resulted in time-resolved spatial *t*-maps, depicting clusters of significant EEG–fMRI correlations, created by Tools for NIfTI and ANALYZE image (Shen, 2014, https://www.mathworks.com/matlabcentral/fileexchange/8797-tools-for-nifti-and-analyze-image).

#### Figures

Figures were created using SPM 12 (Penny et al., 2007), the RainCloud plots (M. Allen et al., 2018, 2021, https://github.com/RainCloudPlots/RainCloudPlots), Inkscape (Inkscape Project, 2020), WFU PickAtlas v3.0 (Maldjian et al., 2003, 2004), MRIcron (Rorden and Brett, 2000, https://www.nitrc.org/projects/mricron, www.mricro.com), and a Colin 27 average brain template (Holmes et al., 1998, http://www.bic.mni.mcgill.ca/ServicesAtlases/Colin27).

### Analyses that did not work

#### Data fusion with the in-scanner EEG

The initial plan for this project was to fuse the fMRI data with the EEG data acquired during scanning. Using the simultaneous EEG data for fusion, the spatiotemporal mapping could then have been performed on a trial-by-trial basis within participants. However, neither a time-resolved RSA nor a perceptual or conceptual LDA classification on the EEG data showed interpretable results, likely due to extensive remaining scanner artifacts in the in-scanner EEG data. As shown previously, head movement during simultaneous fMRI acquisition contributes noise signal in the EEG recordings, particularly within a frequency range under 20 Hz (Fellner et al., 2016). This becomes especially problematic for information decoding, given the importance of signals in the theta band (3–7 Hz) and below for decoding stimulus content (Vidaurre et al., 2021).

#### Data fusion with correlation-based EEG RDMs

Having decided to use the out-of-scanner EEG dataset for the data fusion, we first attempted to perform the fMRI-EEG fusion using correlation-based RDMs calculated from the EEG data (Cichy and Oliva, 2020). To compute these RDMs, we arranged object-specific trials in the same order between all participants, independent of their perceptual format, identical to the arrangement in the fMRI data. We then performed a time-resolved RSA using EEG amplitude values from the 128 electrodes, at a given time bin, as feature vectors. Specifically, we calculated the pairwise distance [1 − correlation (*r*)] between each pair of objects. However, inspecting the average overall similarity and contrasting the average similarity within versus between conceptual classes did not show interpretable results. This is most likely due to too unreliable and time-varying single–trial estimates from the EEG data. Following this initial analysis, we thus used the cross-subject, classification-based RDMs that produced more stable results on the EEG data itself.

## Results

In our memory paradigm, based on Linde-Domingo et al. (2019), participants first encoded novel associations between objects and action verbs. Importantly, object images varied along a perceptual (photographs vs line drawings) and a conceptual (animate vs inanimate) dimension that were used for later classification to distinguish between the two hierarchical processing levels. At the recall stage, following a distracter task, participants were asked to retrieve the object in as much detail as possible when presented with the verb and to indicate the time point of subjective recall with a button press while holding the retrieved image in mind. Following this subjective button press, participants answered either a perceptual (was the object a photograph/drawing?) or conceptual (was the object animate/inanimate?) question on each recall trial. Using this paradigm in an fMRI environment, we aimed at pinpointing the retrieval-related reinstatement of different object features in the brain. A searchlight classification approach was used to spatially locate perceptual (photograph vs drawing) and conceptual (animate vs inanimate) representations during image encoding and retrieval. The next aim was to map spatial representations onto a precise timeline preceding the subjective recall button press on a trial-by-trial basis within subjects, using simultaneous EEG–fMRI data acquisition. Since the within-scanner data turned out to be too noisy to decode the relevant object features (see Materials and Methods, Analyses that did not work), we instead used a previously acquired out-of-scanner EEG dataset (Linde-Domingo et al., 2019) to map the spatial representations onto the memory reconstruction timeline. This was done by means of an EEG–fMRI data fusion, using a second-order RSA (Kriegeskorte et al., 2006, 2008; Kriegeskorte, 2009; Kriegeskorte and Kievit, 2013; Nili et al., 2014).

### Behavior

We first analyzed the behavior of participants who completed the fMRI study and descriptively compared their performance with the participants who took part in the out-of-scanner EEG study (Linde-Domingo et al., 2019). The in- and out-of-scanner encoding data showed comparable RTs and accuracy rates (Table 1). However, looking at subjective retrieval RTs (Fig. 1*c*), participants in the fMRI study were 1.24 s faster on average to indicate subjective retrieval than participants in the EEG study. This RT difference was not only a result of the repeated retrievals in the fMRI group, since RTs of both the first (*M*1 = 1.93 s; SD1 = 0.67 s) and second (*M*2 = 1.58 s; SD2 = 0.66 s) retrieval repetition of the fMRI group were substantially shorter than those of the EEG group. Participants in the fMRI sample might thus have tended to indicate subjective retrieval prematurely, possibly due to discomfort in the scanner environment, and this may have had knock-on effect on the catch question RTs discussed in the next paragraph.

**Table 1.** *M* and SD of encoding and subjective retrieval RTs and accuracies of in- and out-of-scanner data

	In-scanner	Out-of-scanner
Encoding RT	*M *= 3.03 s; SD = 0.90 s	*M *= 2.82 s; SD = 1.56 s
Retrieval RT	*M *= 1.75 s; SD = 0.66 s	*M *= 2.99 s; SD = 0.81 s
Retrieval accuracy	*M *= 84.58%; SD = 0.07	*M *= 86.75%; SD = 0.06

After an explorative inspection of the behavioral data, we compared accuracies and RTs between perceptual and conceptual catch questions using paired-sample *t* tests (Table 2). Note that, different from our previous behavioral work (Linde-Domingo et al., 2019; Lifanov et al., 2021), the paradigm used here was optimized for capturing neural retrieval patterns rather than measuring feature-specific reaction times. After cue onset, participants were first instructed to mentally reinstate the object and indicate retrieval by button press. At this stage, we expected participants to have a fully reconstructed image of the recalled object in mind, such that answering the perceptual and conceptual catch question afterward would take equally long. In the EEG study (Linde-Domingo et al., 2019), participants indeed answered conceptual and perceptual questions equally fast [*t*_(23)_ = 0.5; *p *= 0.62 (unc.)] and accurately [*t*_(23)_ = −1.80; *p *= 0.09 (unc.)]. Participants in the fMRI study, however, performed less accurately [*t*_(30)_ = −6.49; *p *< 0.001 (unc.)] and slower [*t*_(30)_ = 8.47; *p *< 0.001 (unc.)] when answering perceptual compared with conceptual questions (Fig. 1*d*,*e*). The discrepancy between the EEG and fMRI samples, together with the difference in subjective retrieval time, suggests that participants in the fMRI study often pressed the subjective retrieval button before recall was complete, such that the feature reconstruction process carried over into the catch question period (Linde-Domingo et al., 2019; Lifanov et al., 2021).

**Table 2.** *M* and SD of RTs and accuracies for perceptual and conceptual questions separately of (a) in- and (b) out-of-scanner data and paired-sample *t* test between perceptual and conceptual performances (see Extended Data Tables 2-7–2-9 for univariate and Extended Data Tables 2-3–2-6b for multivariate fMRI results)

	RTs	Accuracy
(a) In-scanner		
Perceptual	*M *= 1.30 s; SD = 0.35 s	*M *= 0.82; SD = 0.08
Conceptual	*M *= 1.06 s; SD = 0.25 s	*M *= 0.88; SD = 0.07
*t* test	*t*_(30)_ = 8.47; CI = [0.18, 0.30]; *p *< 0.001 (unc.)	*t*_(30)_ = −6.49; CI = [−0.08, −0.04]; *p *< 0.001 (unc.)
(b) Out-of-scanner		
Perceptual	*M *= 1.38 s; SD = 0.35 s	*M *= 0.86; SD = 0.07
Conceptual	*M *= 1.36 s; SD = 0.30 s	*M *= 0.88; SD = 0.07
*t* test	*t*_(23)_ = 0.5; CI = [−0.06, 0.10]; *p *= 0.62 (unc.)	*t*_(23)_ = −1.80; CI = [−0.05, 0.00]; *p *= 0.09 (unc.)

Despite these RT differences, accuracies were comparably high in the two samples. Moreover, in the EEG–fMRI fusion analyses reported further below, the fMRI data are mainly used to derive the spatial representational patterns, while the precise time resolution is provided by the EEG data (for a review, see Jorge et al., 2014). Considering the sluggishness of the hemodynamic response (Friston et al., 1994; Kruggel and von Cramon, 1999; Glover, 2011), the faster button presses in the fMRI sample should thus only have minimal effects on the fusion results.

### EEG multivariate results

Plots in Extended Data Figure 4-1 show the time-resolved decoding accuracy from the cross-subject classifier, once in gray averaged across all object pairings in the RDM and in magenta split into the pairings belonging to the same (within) or different (between) conceptual classes, all with corresponding statistics (see Materials and Methods).

During encoding, individual object decoding accuracy started increasing gradually from ∼25 ms after the stimulus onset, with a first smaller peak ∼127 ms [*p* < 0.05 (cluster)] and a second temporally extended cluster from 236 ms until the end of the trial [*p* < 0.05 (cluster)]. Moreover, objects from conceptually different classes (animate vs inanimate) were classified significantly better than objects from the same class, starting from 220 ms after the stimulus onset until 1.3 s [*p* < 0.05 (cluster)]. Given the object recognition literature (Cichy et al., 2014), it was surprising that the accuracy of object-identity decoding was comparatively low (though still significant) within the first 100 ms compared with the time period after 200 ms. This might reflect the fact that across participants, the objects corresponded in terms of image content but not perceptual format, potentially suppressing some low-level similarities and thus making classification on early visual features more difficult. That said, the photographic and drawn object versions overlapped in shape, orientation, and disparity, preserving at least some low-level features, and a classifier should thus be able to use the neural signals corresponding to these features to discriminate individual objects. In fact, the searchlight fusion shows major correlation clusters during encoding in early visual areas (Fig. 5*a*), indicating that some low-level features contribute to decoding performance at these early time points.

During retrieval, overall cross-subject classification accuracies remained relatively low in comparison with encoding, showing a peak decoding accuracy of 51% approximately −2.8 s before the retrieval button press. Five significant accuracy clusters were found with peaks located at times −2.74, −2.63, −2.56, −2.16, and −1.47 s relative to the time of subjective recollection [all *p* < 0.05 (cluster)]. When comparing classification accuracy for conceptually different, between-class object pairs to within-class pairs, this comparison survived cluster correction at no time point. The maximum between- versus within-class difference was identified about −2.5 s before the retrieval button press, though at a very liberal threshold [*p* < 0.01 (unc.)].

### fMRI multivariate results

**Figure 2.**
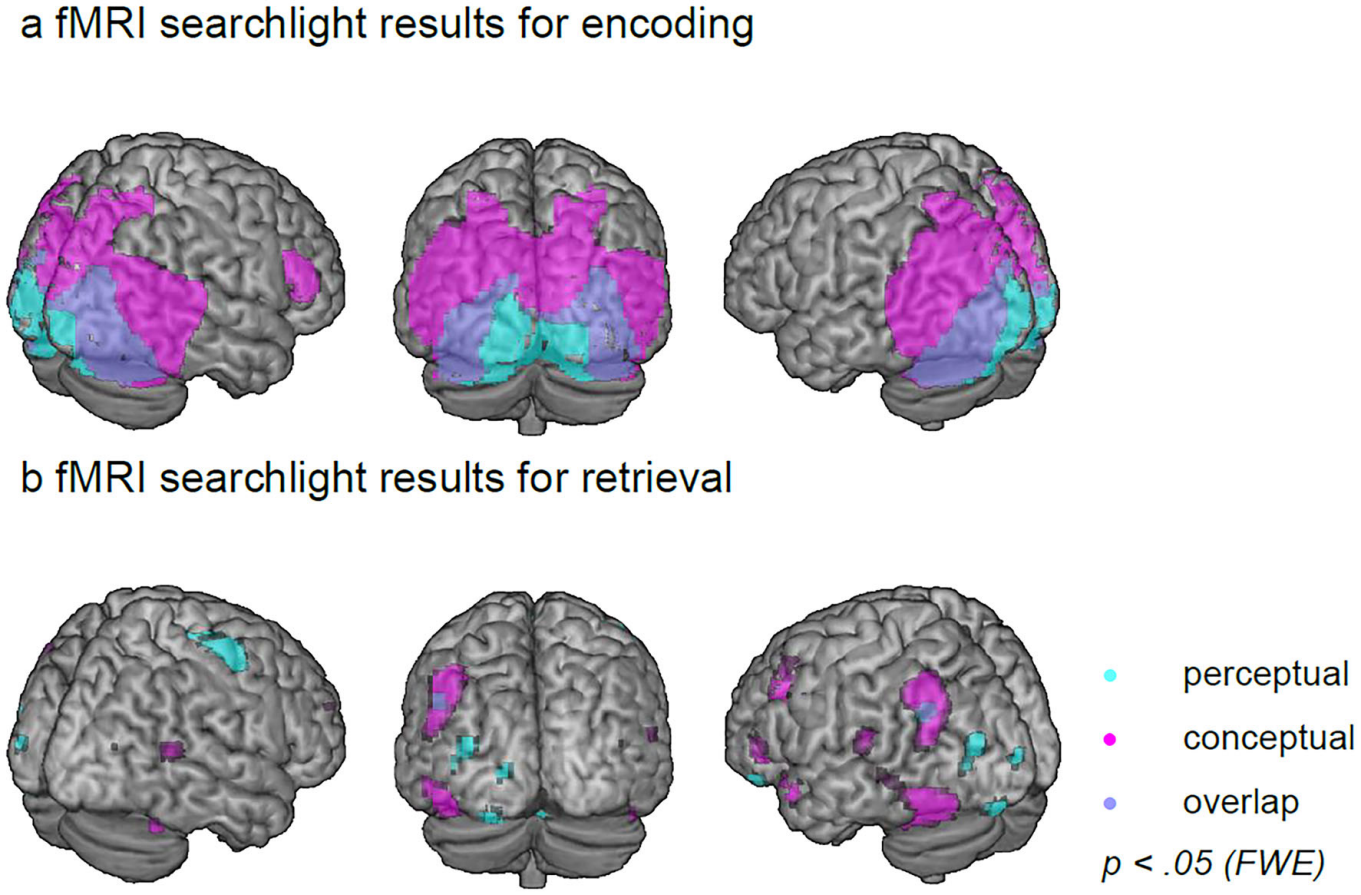
Searchlight LDA results. Second-level *t*-contrasts show (***a***) encoding and (***b***) retrieval accuracies significantly higher than the 50% chance level when classifying perceptual (cyan) and conceptual (magenta) object features; overlap is indicated in purple. All contrasts are thresholded at *p* < 0.05 (FWE-corrected). *N* = 31 independent subjects. Figure made using MRIcron (https://www.nitrc.org/projects/mricron, www.mricro.com, Rorden and Brett, 2000) and a Colin 27 average brain template (http://www.bic.mni.mcgill.ca/ServicesAtlases/Colin27, Holmes et al., 1998; Copyright (C) 1993–2009 Louis Collins, McConnell Brain Imaging Centre, Montreal Neurological Institute, McGill University). See Extended Data Figures 2-1 and 2-2 and Extended Data Tables 2-3–2-9 for more details.

Previous work suggests that during memory recall, conceptual information becomes accessible earlier than perceptual information, as evidenced in neural pattern decodability (Linde-Domingo et al., 2019) and behavior (Lifanov et al., 2021; Linde-Domingo et al., 2019; and in this paper, Fig. 1*d*). The first aim of the present study was to determine the locus of these perceptual and conceptual representations both during encoding and retrieval. We thus performed a searchlight-based LDA. Two separate classifiers were trained to distinguish between the different perceptual (photographs vs drawings) and the different conceptual (animate vs inanimate) classes of our visual objects (see Materials and Methods for details). During encoding (Fig. 2*a*), when the object was present on the screen, perceptual class could be decoded in posterior regions along the ventral visual stream including V1, V2, lingual, and fusiform gyrus [*t*_(30)_ = 4.98; *p *< 0.05 (FWE)]. Conceptual class was decodable from more anterior lateral temporal lobe areas, including inferior, mid, and superior temporal gyrus, and from the precuneus and the inferior frontal gyrus, dorsolateral prefrontal cortex, and anterior prefrontal cortex [*t*_(30)_ = 4.82; *p *< 0.05 (FWE)]. Together, this mapping generally reflects a posterior-to-anterior perceptual-to-conceptual processing gradient. The results largely mirror the univariate analyses (Extended Data Fig. 2-2) and complement them by showing additional content decoding in frontal and parietal regions, suggesting that these areas code information in fine-grained multivoxel patterns that univariate analyses are unable to capture.

10.1523/ENEURO.0091-24.2024.t2-1 Figure 2-1 Searchlight LDA results illustrating encoding-retrieval overlap. Second-level t-contrasts show a) perceptual and b) conceptual accuracies significantly higher than the 50% chance level when classifying from the encoding (cyan/magenta) and retrieval (dark blue/red) data. All contrasts are thresholded at p < .05 (FWE-corrected). N = 31 independent subjects. Figure made using MRIcron (https://www.nitrc.org/projects/mricron, www.mricro.com, Rorden & Brett, 2000) and a colin 27 average brain template (http://www.bic.mni.mcgill.ca/ServicesAtlases/Colin27, Holmes et al., 1998; Copyright (C) 1993–2009 Louis Collins, McConnell Brain Imaging Centre, Montreal Neurological Institute, McGill University). Download Figure 2-1, TIF file.

10.1523/ENEURO.0091-24.2024.f2-2 Figure 2-2 Univariate results. Second-level t-contrasts for encoding. a) Cyan: Photograph > drawing, dark blue: drawing > photograph. b) Magenta: animate > inanimate, yellow: inanimate> animate. All contrasts are thresholded at t(30) = 4.56, p < .05 (FWE). N = 31 independent subjects. During retrieval, no cortical voxels survived family-wise error correction (p < .05 FWE) when contrasting perceptual or conceptual categories. Figure made using MRIcron (https://www.nitrc.org/projects/mricron, www.mricro.com, Rorden & Brett, 2000) and a colin 27 average brain template (http://www.bic.mni.mcgill.ca/ServicesAtlases/Colin27, Holmes et al., 1998; Copyright (C) 1993–2009 Louis Collins, McConnell Brain Imaging Centre, Montreal Neurological Institute, McGill University). Download Figure 2-2, TIF file.

10.1523/ENEURO.0091-24.2024.t2-3 Table 2-3 fMRI searchlight results for encoding: perceptual features. Download Table 2-3, DOC file.

10.1523/ENEURO.0091-24.2024.t2-4 Table 2-4 fMRI searchlight results for encoding: conceptual features. Download Table 2-4, DOC file.

10.1523/ENEURO.0091-24.2024.t2-5 Table 2-5 fMRI searchlight results for retrieval: perceptual features. Download Table 2-5, DOC file.

10.1523/ENEURO.0091-24.2024.t2-6 Table 2-6 fMRI searchlight results for retrieval: a) conceptual features and b) conceptual features > perceptual features. Download Table 2-6, DOC file.

10.1523/ENEURO.0091-24.2024.t2-7 Table 2-7 fMRI univariate results for encoding: photos > drawings. Download Table 2-7, DOC file.

10.1523/ENEURO.0091-24.2024.t2-8 Table 2-8 fMRI univariate results for encoding: animate > inanimate. Download Table 2-8, DOC file.

10.1523/ENEURO.0091-24.2024.t2-9 Table 2-9 fMRI univariate results for encoding: inanimate > animate. Download Table 2-9, DOC file.

During retrieval (Fig. 2*b*), perceptual features were most strongly decodable in the precentral gyrus (i.e., the premotor cortex) but also in the precuneus, angular gyrus, cingulate cortex, inferior frontal gyrus, and ventral areas including V2, fusiform gyrus, and middle temporal lobe [*t*_(30)_ = 4.80; *p *< 0.05 (FWE)]. Conceptual category membership was classified with the highest accuracy from fusiform gyrus, middle temporal gyrus, temporal pole, precuneus, angular gyrus, dorsolateral prefrontal cortex, and inferior and superior frontal gyrus [*t*_(30)_ = 4.60; *p *< 0.05 (FWE); for detailed LDA results, see Extended Data Tables 2-3–2-6*b*]. Hence, in addition to the ventral visual areas dominating encoding/perception, we also found extensive frontal and parietal areas in our retrieval searchlight, in line with previous work suggesting that mnemonic as opposed to sensory content can often be decoded from these areas (Kuhl and Chun, 2014; Favila et al., 2018, 2020; Ferreira et al., 2019). At liberal thresholds, a paired *t* test (Extended Data Table 2-6*b*) contrasting perceptual versus conceptual classifications during retrieval showed higher conceptual classifications in the frontal [*t*_(30)_ = 3.46; *p* = 0.001 (unc.)], dorsolateral prefrontal cortex [*t*_(30)_ = 3.28; *p* = 0.001 (unc.)], temporal pole [*t*_(30)_ = 2.71; *p* = 0.005 (unc.)], and precuneus [*t*_(30)_ = 2.47; *p* = 0.01 (unc.)]. Paired *t* tests at the same liberal thresholds showed no higher accuracies for perceptual decoding during retrieval. Together, the fMRI-based classification of the two dimensions that we explicitly manipulated demonstrates that brain activity patterns during the retrieval of object images carry information about perceptual format and conceptual content, with the latter dominating the recall patterns. Some areas partially overlapped with the areas seen at encoding, but retrieval additionally comprised unique contributions in frontoparietal networks (overlap visualized in Extended Data Fig. 2-1). Note, also, that regional overlap in content decodability in this analysis does not necessarily imply and exact pattern overlap between encoding and retrieval.

No voxels survived (at *p* < 0.05, FWE-corrected) when cross-classifying perceptual or conceptual categories from encoding to retrieval or from retrieval to encoding, which might be due to the overall noisier retrieval patterns, or due to a transformation of these category representations between encoding and retrieval (see Discussion).

### EEG–fMRI data fusion

#### EEG multivariate analysis

**Figure 3.**
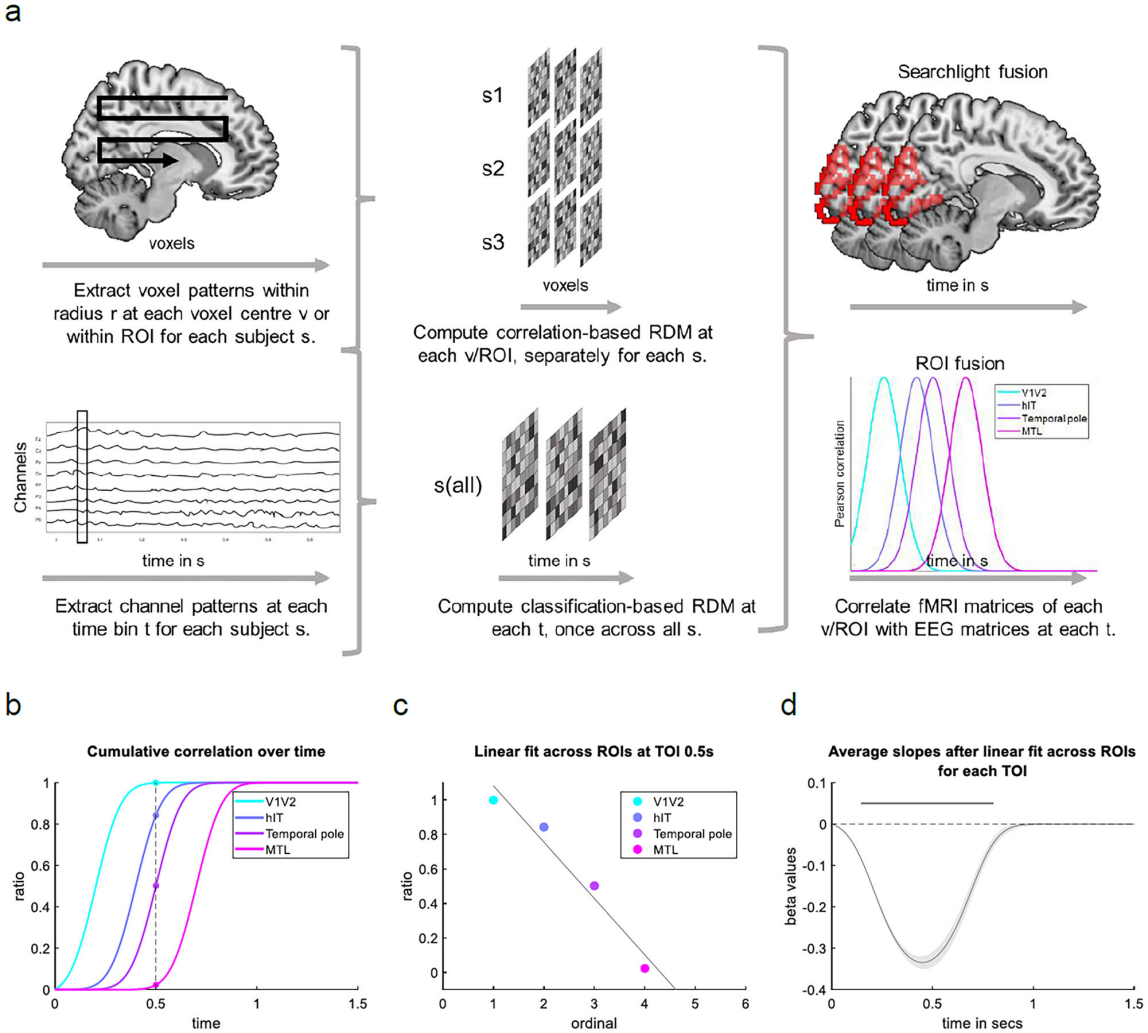
***a***, Overview of ROI and searchlight fusion. First, brain activity patterns are extracted from fMRI and EEG to create RDMs in the spatial and temporal domain, respectively. Then, second-order correlations of the RDMs from the two imaging modalities are computed for the data fusion. The EEG RDMs are classification-based, derived from binary classifiers trained and tested to distinguish each pair of objects across participants. FMRI RDMs are computed within participants and are correlation-based. FMRI RDMs can be computed at each voxel center (v) including voxels within a specific radius or for larger ROI. Depending on this choice, the data fusion will be searchlight- or ROI-based, respectively. ***b***, ***c***, Linear regression approach on ROI time courses in a forward stream simulation. ***b***, Normalized cumulative sum of each ROI time course of the ventral visual stream. At time point 0.5 s, earlier visual regions show a higher cumulative sum than later regions along the ventral visual stream. ***c***, A linear regression is fitted across ROI values at 0.5 s and results in a negative slope. ***d***, Average slopes and standard errors are illustrated for each time point across the window 0–1.5 s and tested against zero. Significant time points are indicated by points (*p* < 0.01 unc.). Method and panels ***b–d*** adapted from Michelmann et al. (2019). Brain figures made using MRIcron (https://www.nitrc.org/projects/mricron, www.mricro.com, Rorden and Brett, 2000), a Colin 27 average brain template (http://www.bic.mni.mcgill.ca/ServicesAtlases/Colin27, Holmes et al., 1998; Copyright (C) 1993–2009 Louis Collins, McConnell Brain Imaging Centre, Montreal Neurological Institute, McGill University) and WFU PickAtlas v3.0 (Lancaster et al., 1997, 2000; Tzourio-Mazoyer et al., 2002; Maldjian et al., 2003, 2004).

The next goal of this study was to map the spatial patterns in a given brain area onto the EEG patterns present at each time point of a trial, to reveal how content representations evolve over the time course of encoding and retrieval. For this data fusion, we first needed to obtain a meaningful EEG matrix representing object-to-object similarities for each time point of encoding and retrieval, which we could then compare with the fMRI-based matrices. Correlation-based RDMs for the EEG data were too noisy to see a meaningful match between modalities (see Materials and Methods, Analyses that did not work). We thus created a classification-based matrix representing the dissimilarities between each pair of objects, in each EEG time bin, based on a cross-participant classifier. Amplitudes over the 128 electrodes at a given time point served as classification features, and our 24 participants provided the repetitions of each object. The pairwise classification accuracies were entered in a single, time-resolved RDM structure representing the dissimilarity between individual objects of our stimulus pool collapsed across participants (Fig. 3*a*). For all fusion analyses, we investigated encoding timelines from the image onset and retrieval timelines leading up to the subjective button press.

#### ROI fusion

Having obtained this EEG-based similarity time course, we performed a similarity-based fusion of the EEG and fMRI data, probing what brain regions (in the fMRI data) most closely matched the similarity structures contained in the EEG data at any given time point during encoding and retrieval. For a first fusion step, we used the representational geometries (i.e., the RDMs) from an anatomically predefined set of ROIs. We then calculated the correlation between these ROIs’ representational geometries and the geometries obtained from the EEG data for each time bin (Fig. 3*a*) and statistically compared the correlations against zero using cluster-basted permutation testing.

To statistically evaluate whether the information flow is primarily forward or backward along ventral visual stream areas, we used a linear regression on the cumulative sums of the correlation time courses of the corresponding ROIs, a method previously established for MEG analyses (Michelmann et al., 2019). The intuition of this method is that if Region 1 starts accumulating information before Region 2 and Region 2 before Region 3, the cumulative sums will line up sequentially to reflect this systematic delay in information accumulation (Fig. 3*b*–*d*, simulation). In our case, we expected forward progressing latencies along the ventral visual stream during encoding and backward progressing latencies during retrieval (Fig. 3*b*–*d*).

**Figure 4.**
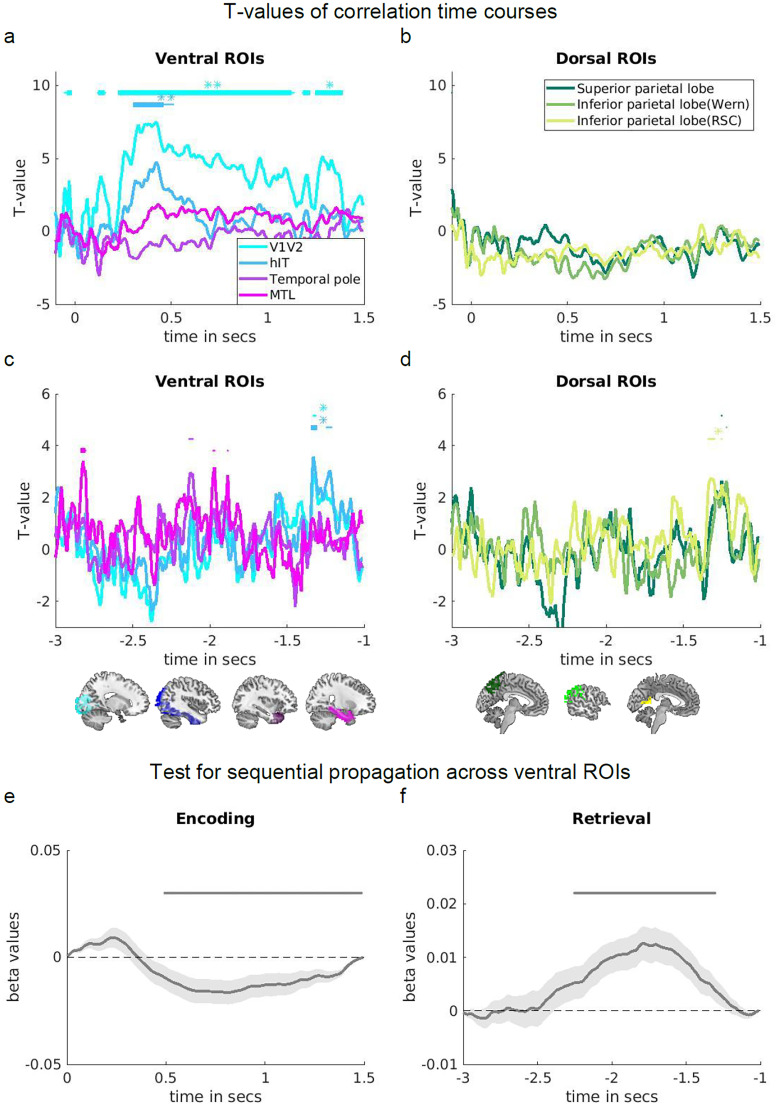
***a–d***, *T* values from one-sample *t* test of correlation time courses between the EEG RDMs and ventral visual (***a***, ***c***) and dorsal (***b***, ***d***) ROIs during encoding (***a***, ***b***) and retrieval (***c***, ***d***). ROIs are color-coded as indicated in the legends. Significant time points are indicated by points (*p* < 0.01 uncorrected), squares (*p* < 0.01 Bonferroni corrected for multiple comparison of 7 ROIs), one asterisk (*p* < 0.05 cluster), and two asterisks (*p* < 0.05 cluster, Bonferroni corrected for multiple comparison of 7 ROIs). ***e***, ***f***, Results of the test for sequential processing, using a linear fit of the cumulative sums of correlation time courses across ROIs within the ventral visual stream at (***e***) encoding and (***f***) retrieval. Average slopes and standard errors are shown for each time point and statistically tested against zero. A negative slope suggests that earlier ROIs along the ventral visual stream have a higher cumulative sum than later ROIs, indicative of a forward stream. According to the same logic, a positive slope indicates a backward stream. Significant time points are indicated by gray points above the curve (*p* < 0.05 cluster). Method adapted from Michelmann et al. (2019). In all subpanels, time point 0 s marks the object onset during encoding and the subjective recall button press during retrieval. The latter is not included on time axis as it does not lie within the time window of interest (see Materials and Methods also for details on cluster correction). Variance in all subpanels comes from *n* = 31 independent subjects. Brain figures made using MRIcron (https://www.nitrc.org/projects/mricron, www.mricro.com, Rorden and Brett, 2000), a Colin 27 average brain template (http://www.bic.mni.mcgill.ca/ServicesAtlases/Colin27, Holmes et al., 1998; Copyright (C) 1993–2009 Louis Collins, McConnell Brain Imaging Centre, Montreal Neurological Institute, McGill University), and WFU PickAtlas v3.0 (Lancaster et al., 1997, 2000; Tzourio-Mazoyer et al., 2002; Maldjian et al., 2003, 2004). See Extended Data Figures 4-1 and 4-2 for more details.

Our predefined set of ROIs included regions along the ventral visual stream (V1/V2, hIT, temporal pole, and MTL), and regions along the dorsal stream (superior parietal lobe, lateral inferior parietal cortex, and medial parietal cortex; see Materials and Methods, ROIs). Since many computational models propose that the role of the hippocampus is related to association or indexing rather than feature representation (Teyler and DiScenna, 1986; McClelland et al., 1995; Eichenbaum, 2001; O’Reilly and Norman, 2002; Rolls, 2010, 2013), we excluded the hippocampus from the MTL regions. In Figure 4*a–d*, correlation time courses are plotted for each individual ROI as *t* values resulting from *t* tests against zero, with clusters resulting from a cluster-based permutation test (see Materials and Methods, Analyses). The figure shows ventral visual regions in panel *a*/*c* and dorsal regions in panel *b*/*d*. Note that in-text significance statements give information about uncorrected *p* values (unc.), *p* values Bonferroni’s corrected for multiple comparisons (Bonferroni’s, 1936), temporal clusters, or temporal clusters additionally corrected for multiple comparisons.

10.1523/ENEURO.0091-24.2024.f4-1 Figure 4-1 Average accuracy of EEG-based classification of object identity over time (grey), and average classification accuracy within- versus between conceptual classes (magenta) during a) encoding and b) retrieval. At encoding, time point 0 s marks the object onset. At retrieval, time point 0 s marks the button press, but is not included in figure as it does not lie within the time window of interest (see methods). Solid grey line represents average accuracy across the entire RDM, dashed grey line represents baseline average, and grey markers indicate significant overall classification accuracy against chance through random label permutation, compared by independent-sample t-tests. Solid magenta line represents the average accuracy within conceptual categories, dashed magenta line represents average accuracy between conceptual categories, and magenta markers indicate significant between- versus within conceptual classes with correct labels, compared by paired-sample t-tests. Significant decoding accuracy is indicated by points (p < .01 unc.) and asterisk (p < .05 cluster). See Methods for details on cluster correction. N = 24 independent subjects. Download Figure 4-1, TIF file.

10.1523/ENEURO.0091-24.2024.f4-2 Figure 4-2 Region of interest (ROI) fusion: linear fit. A-b) Average cumulative sums of correlations and standard error (in zoomed in version) across subjects within a) encoding and b) retrieval. Dashed vertical line indicates the time point of minimum beta value for encoding (0.81 s) and maximum beta value for retrieval (-1.79 s). c-d) A linear fit of the cumulative sums of correlation time courses across ROIs within the ventral visual stream. A linear fit was made for an exemplar TOI (time of interest), 0.81 s for encoding c) and -1.79 s for retrieval d). Plots showing average ratio (thick dots) and average slope (thick line), as well as ratio and slopes within participants (small dots, thin lines). ROIs are color-coded as in legend. A negative slope suggests that earlier ROIs along the ventral visual stream have a higher cumulative sum than later ROIs, indicative of a forward stream. According to the same logic, a positive slope indicates a backward stream. Method adapted from Michelmann et al., 2019. In a-b), time point 0 s marks the object onset during encoding, and the subjective recall button press during retrieval. The latter is not included on time axis as it does not lie within the time window of interest (see Methods, also for details on cluster correction). Variance comes from n = 31 independent subjects. Download Figure 4-2, TIF file.

Following the object onset at encoding (Fig. 4*a*), posterior regions including V1 and V2 showed an increasing correlation with the EEG representations from ∼120 ms [*p* < 0.01 (mc corr.)]. A significant cluster was seen from 240 ms onward and remained active for ∼1 s [*p* < 0.05 (corr. cluster)], followed by another cluster at 1.31 s after the image onset [*p* < 0.05 (unc. cluster)]. The first cluster was followed by a correlation increase of later ventral visual areas (the hIT cortex) ∼310 ms, reaching a significant peak at ∼420 ms [*p* < 0.05 (corr. cluster)]. Later lateral and medial temporal as well as parietal regions (Fig. 4*b*) did not show a significant correlation with the EEG time series during encoding.

As a proof of principle, we then tested for a forward processing stream in the encoding data across ventral visual areas. Performing a linear regression on the cumulative sums of all ventral ROI time courses at each time point (see Materials and Methods, Fig. 3*b*–*d*, Extended Data Fig. 4-2*a*,*c*), we found negative slopes reaching significance 500 ms after the stimulus onset [*p* < 0.05 (cluster)]. This finding statistically corroborates the observation that earlier ventral visual regions code relevant information before later regions, supporting the well established forward stream (Fig. 4*e*). It should be noted, however, that evidence for such feedforward processing was only present relatively late in the trial, likely since the strongest peaks of object-identity decoding also fell in this late time window (Extended Data Fig. 4-1*a*).

When performing the same analysis for ventral visual ROIs during retrieval (Fig. 4*c*), several regions peaked below the threshold of cluster significance: MTL regions showed a peak correlation with the EEG representational geometries −2.96 s [*p* < 0.01 (mc corr.)] and −2.82 and −1.97 s prior to the retrieval button press [*p* < 0.01 (unc.)]. Furthermore, correlation peaks were found for the temporal pole at −2.13 s [*p* < 0.01 (unc.)]. HIT showed a peak correlation with the EEG geometry at −1.33 s [*p* < 0.01 (mc corr.)] and −1.22 s [*p* < 0.01 (unc.)], forming a cluster at −1.27 s [*p* < 0.05 (unc. cluster)]. Similarly, V1/V2 peaked at −1.33 s [*p* < 0.01 (unc.)], showing a cluster at −1.27 s before button press [*p* < 0.05 (unc. cluster)].

Within the dorsal set of ROIs (Fig. 4*d*), the inferior medial parietal lobe showed a correlation peak in a similar time window, at −1.33 s before button press [*p* < 0.05 (unc. cluster)]. This and the peak of the inferior lateral parietal ROI at −1.22 s [*p* < 0.01 (unc.)], as well as superior parietal ROI at −1.25 s [*p* < 0.01 (unc.)], coincided with the peak times of posterior ventral visual areas (V1/V2/hIT).

Our main aim of the data fusion was to test if memory reactivation followed a backward processing hierarchy along the ventral visual stream. We therefore also performed a linear regression on cumulative sums of ventral visual regions within retrieval (Extended Data Fig. 4-2*b*,*d*). This analysis revealed a significant positive slope from −2.2 to −1.3 s before button press [*p* < 0.05 (cluster)], speaking in favor of a backward stream and thus confirming our main hypothesis (Fig. 4*f*).

#### Searchlight fusion

**Figure 5.**
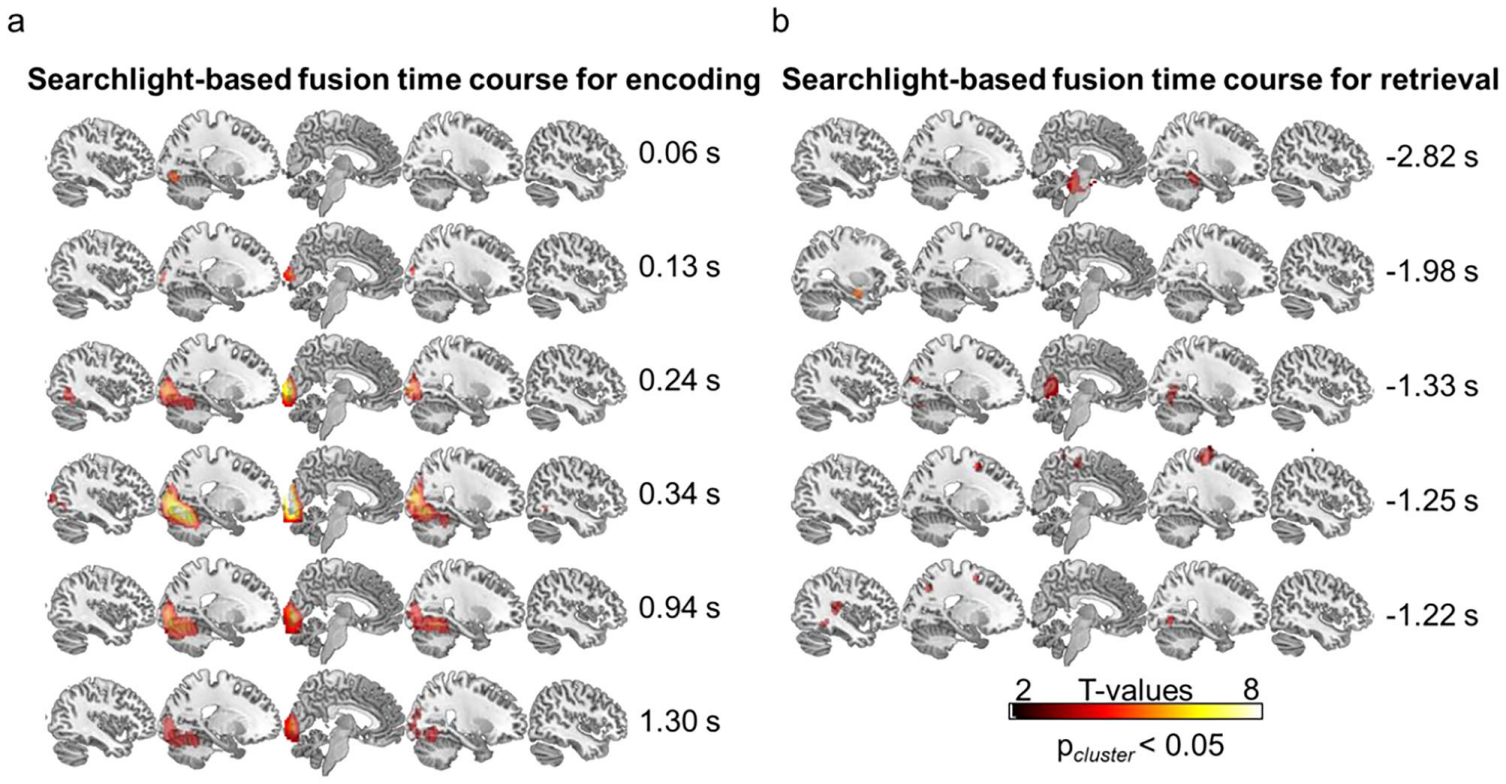
*T*-maps from one-sample *t* test of EEG–fMRI correlations at (***a***) encoding and (***b***) retrieval showing time points in the trial time course where significant spatial clusters of correlations emerged (*p*_cluster _< 0.05; see Materials and Methods for further details). *T* test involved a spatial maximal permuted statistic correction combined with a threshold-free cluster enhancement (Nichols and Holmes, 2002; Smith and Nichols, 2009). At encoding, time point 0 s would mark the object onset. At retrieval, time point 0 s marks the button press; however, both zero time points are not included in figure as no clusters emerged. *N* = 31 independent subjects. Brain figures made using MRIcron (https://www.nitrc.org/projects/mricron, www.mricro.com, Rorden and Brett, 2000) and a Colin 27 average brain template (http://www.bic.mni.mcgill.ca/ServicesAtlases/Colin27, Holmes et al., 1998; Copyright (C) 1993–2009 Louis Collins, McConnell Brain Imaging Centre, Montreal Neurological Institute, McGill University).

Next, in a more explorative but also spatially better resolved approach, we conducted a whole-brain searchlight fusion to inspect where across the brain the fMRI-based representational geometries matched the EEG geometries over time. Figure 5 depicts spatial *t*-maps representing significant EEG–fMRI correlations in each searchlight radius for a given time bin, separately at encoding (Fig. 5*a*) and retrieval (Fig. 5*b*). The maps only show time points where significant spatial clusters emerged. During encoding, the searchlight fusion revealed a significant cluster in V1 and lingual gyrus at 60 ms following the object onset and again from 130 to 160 ms and from 240 ms onward, reactivating several more times (e.g., see timepoints 940 ms and 1.3 s) before fading ∼1.4 s after the stimulus onset. Importantly, over the time course of object perception, significant clusters of EEG–fMRI similarity gradually extended from early visual (V1, V2) to more ventral and lateral areas, including fusiform and inferior temporal gyrus. For the encoding data, the results of the searchlight approach thus overlap with the ROI fusion results and mainly reveal ventral visual stream activation progressing in a forward (early-to-late visual cortex) manner.

During retrieval, the earliest cluster of significant EEG–fMRI correlations was found in the fusiform area, preceding the subjective retrieval button press by −2.82 s, accompanied by a small cluster in the pons. Contrary to our prediction, we also observed pattern overlap in the hippocampus at −1.98 s before button press.

At time points closer to the button press, additionally clusters showing significant pattern correlations were found in lower-level regions. At −1.33 s, V1, V2, V4d, and the fusiform gyrus showed significant clusters. Furthermore, at −1.25 s, clusters were found in the premotor cortex, primary motor cortex, and superior parietal lobule (SPL). Finally, clusters were found in the premotor cortex, SPL, primary and associative visual cortex, fusiform area, and Wernicke's area at −1.22 s before subjective retrieval.

Together, the retrieval data showed convergence between the ROI and searchlight fusion approach. It showed a progression of the peak correlations from frontotemporal to posterior sensory and parietal regions, generally reflecting a backward propagating processing stream which, however, only partially revisits the areas involved during encoding, thus suggestive of substantial transformations between object perception and memory reconstruction.

## Discussion

What mnemonic content is recovered, where in the brain during memory retrieval, and how does the hippocampal–neocortical pattern completion process unfold in time? Recent memory research suggests that the information processing hierarchy is reversed during the recall of a visual object from episodic memory compared with its initial perception (Linde-Domingo et al., 2019; Lifanov et al., 2021; Mirjalili et al., 2021), with conceptual features becoming available earlier than perceptual features. Here, we investigated the locus of these feature representations during encoding and recall using fMRI-based decoding. Additionally, EEG–fMRI fusion allowed us to test whether this presumed reversed information processing cascade during memory reconstruction maps onto the same ventral visual stream areas that carry the information forward during perception but now following a backward trajectory. Our study shows that recall is largely dominated by conceptual information in the posterior ventral regions and that the reconstruction of visual object memories, while following a backward trajectory along ventral visual object processing pathways to some extent, also involves an information relay to parietal and frontal regions.

We first used uni- and multivariate analyses on the fMRI data alone to map out the regions processing perceptual and conceptual object dimensions that were built into the stimulus set. As expected, during encoding when the object was visible on the screen, these features largely mapped onto the ventral visual pathway, where early visual areas coded the perceptual features (colored photos vs black-and-white line drawings), whereas later visual areas coded the mid- to higher-level conceptual information (animate vs inanimate objects; B. Long et al., 2018). This general perceptual-to-conceptual gradient is in line with a multitude of findings in the basic object recognition and vision literature (Carlson et al., 2013; Kravitz et al., 2013; Cichy et al., 2014).

Univariate analyses were not powerful enough to detect differential activations between perceptual and conceptual categories when objects were reconstructed from memory. Activity patterns at the finer-grained multivoxel level, however, carried information about the categorical features of the retrieved images. Memory-related reactivation of these features comprised some regions also found during object encoding, particularly in late visual areas (Fig. 2*b*, Extended Data Fig. 2-1). This partial overlap confirms a body of previous work showing that posterior visual areas are not only involved in visual perception but also in internally generated processes, such as mental imagery (Kosslyn et al., 1993; Dijkstra et al., 2021) or episodic memory. While most memory studies find reactivation in late areas along the ventral stream (Polyn et al., 2005; Staresina et al., 2012; Griffiths et al., 2019), others have reported areas as early as V1 (Bosch et al., 2014). It is also commonly observed that stronger reinstatement is associated with memory success and strength (Huijbers et al., 2011; Staresina et al., 2012; Ritchey et al., 2013; Wing et al., 2014; Thakral et al., 2015) and the vividness and detail of remembering (Wheeler et al., 2000; St-Laurent et al., 2015; Bone et al., 2020; Simons et al., 2022), suggesting an important functional role of sensory reactivation. Assuming a hierarchical reverse processing from higher to lower sensory regions during recall, incomplete retrieval would lead primarily to a loss of perceptual information. Premature retrieval button presses, as found in the present study (see behavioral results), could indicate such incomplete recall and explain the overall weaker decodability of perceptual features.

Although the spatial localizations of feature-specific patterns during recall partly overlapped with those found during encoding, they also encompassed regions outside the ventral visual object processing pathways. Most notably, conceptual object information during recall was decodable from frontal and parietal regions. The role of the frontoparietal cortex in representing mnemonic information is still debated (Buckner et al., 1999; Kramer et al., 2005; Levy, 2012; Favila et al., 2018, 2020; Staresina and Wimber, 2019; Humphreys et al., 2021; Xue, 2022). Some studies suggest that depending on the task and functional state (i.e., encoding or retrieval), content representations are preferentially expressed in sensory or parietal cortices, respectively, and mediated by differential connectivity with the hippocampus (Favila et al., 2018; N. M. Long and Kuhl, 2021; for a related review about the posterior medial network, see Ritchey and Cooper, 2020). Others propose that parietal areas provide a contextualization of the retrieved memories (Jonker et al., 2018). Our findings support the previously described encoding–retrieval distinction and suggest that it is predominantly conceptual information (Fig. 2*b*, Extended Data Table 2-6*b*) that is represented in parietal regions during retrieval, in line with previous work showing strong object identity—and even abstract concept coding in the parietal cortex (Jeong and Xu, 2016; Ferreira et al., 2019; Kaiser et al., 2022). The involvement of parietal networks is also discussed further below in relation to the timing of the reactivation.

Going beyond a purely spatial mapping of perceptual and conceptual representations, fusing the EEG and fMRI data allowed us to inject time information into these spatial maps and to ask how the memory reconstruction stream evolves, building up to the time of subjective recollection. We used two complementary approaches for data fusion, both comparing the representational geometries found in the EEG patterns at each time point with the fMRI geometries found in a given brain region. Both the ROI-based and searchlight-based approach showed a mainly feedforward sweep of information processing during the first few hundred milliseconds of object perception, starting within early visual regions ∼60 (searchlight fusion) to 120 ms (ROI fusion) after the image onset and spreading to more ventral visual regions within the next 200 ms (see Materials and Methods, Searchlight fusion and ROI fusion). Interestingly, we observed a sustained response in early visual regions, which we believe to partially be a consequence of the task demand that included visualizing the object for encoding purposes. Importantly, a formal regression analysis allowed us to statistically corroborate the spatiotemporal direction of information flow, by modeling the information accumulation rate across the hierarchy of ventral visual stream regions (Michelmann et al., 2019). Note that while this regression is a powerful method for detecting serial processes across participants (Michelmann et al., 2019), it merely speaks to the order of patterns seen in the ROI time courses, irrespective of the strength and significance of these ROIs’ average peaks. The method should thus be regarded as complementing the ROI-based and searchlight-based fusion analyses. During encoding, later ROI peaks did not show significant correlations, likely due to less time-locked nature of late EEG peak, leading to comparatively smaller mapping effects in the fusion (for comparison, see Cichy et al., 2014, 2016). That said, the results statistically support a posterior-to-anterior progression of the correlation patterns across participants during early phases of the encoding trials, likely driven disproportionally by V1/V2 and hIT patterns.

Similarly, the EEG–fMRI fusion and sequence analyses were applied to the retrieval data. We acknowledge that the retrieval EEG–fMRI correlations were comparatively weak and would, in the case of the fusion analysis, not survive an additional correction for multiple comparisons over time on top of the spatial cluster-based correction. That said, the temporal evolution of effects still offer insights into the memory reconstruction process. The ROI data fusion and linear regression hint at a largely backward information processing trajectory, with information flowing from MTL (both fusion approaches) and temporal pole (ROI fusion) to more posterior visual regions (both fusion approaches). The temporal pole has previously been associated with processing of high-level semantic information (Noppeney and Price, 2002; Patterson et al., 2007; Visser et al., 2010; Rice et al., 2018; Clarke, 2020). Moreover, temporal pole reactivation around −2.1 s before button press was close to the time point when objects from different conceptual classes were more discriminable than objects from similar conceptual classes based on the EEG classification alone (Extended Data Fig. 4-1*b*). Note that our decoding peaks were found considerably earlier than in previous work (Linde-Domingo et al., 2019), likely due to the cross-participant object–identity decoding approach (for comparison, see Linde-Domingo et al., 2019; Mirjalili et al., 2021) which may rely on higher-level exemplar–level information presumably processed close to the hippocampus (Clarke and Tyler, 2015). Together with our previous work using feature-specific reaction times (Linde-Domingo et al., 2019; Lifanov et al., 2021), these findings indicate that conceptual object features in semantic networks are among the first to be reactivated during memory retrieval, which might be indicative of a semanticization process introduced by retrieval (Lifanov et al., 2021).

More posterior areas including inferior temporal, inferior parietal, and visual cortices, as well as frontal regions, reached their representational correlation peaks considerably later, from −1.33 s before button press onward (Figs. 4*c*,*d*, 5*b*). While visual activations can be assumed to represent the perceptual content of the reactivated memories, the role of fronto-/parietal regions, as mentioned earlier, is still a matter of debate (Naghavi and Nyberg, 2005; Barry and Maguire, 2019; Fischer et al., 2021). In addition to the potential semanticization process discussed in the previous paragraph, parietal areas have been shown to play a role in contextual processing, imagery during recall, and scene construction (Fletcher et al., 1995; Lundstrom et al., 2005; Chrastil, 2018). The latest reactivations might thus be indicative of a final stage of recall leading up to subjective recollection, likely involving working memory and memory-related imagery and, hence, preparation for the upcoming categorization task (Ganis et al., 2004; Christophel et al., 2012, 2017). In summary, contrary to encoding, we find that retrieval follows a backward propagation along the ventral visual stream. Moreover, the backward reconstruction flow is not limited to ventral visual brain areas that dominate the encoding patterns but instead involves frontal and parietal regions that may serve as an episodic memory or imagery buffer for the retrieved representations (Baddeley, 1998, 2000; Wagner et al., 2005; Levy, 2012), speaking in favor of a transformative process.

The anterior-to-posterior retrieval stream shown here is reminiscent of a similar reversed stream for object imagery in the absence of an episodic cue (Dijkstra et al., 2020), which has also been described as progressing in a hierarchical fashion along the ventral stream (Horikawa and Kamitani, 2017). Generating mental images from general knowledge compared with cuing them from episodic memory likely involve different neural sources, in the latter case most notably the hippocampus. However, both processes rely on the internal generation of stored mental representations (i.e., memories), and it is thus perhaps not surprising that mental imagery and episodic retrieval overlap in many aspects, including their reverse reconstruction gradient. While there have been various studies comparing imagery with perception (Horikawa and Kamitani, 2017; Dijkstra et al., 2020; Xie et al., 2020), it will be interesting for future studies to provide a more detailed picture of where in the processing hierarchy imagery and memory retrieval overlap.

The fMRI classification and the RSA-based EEG–fMRI fusion in this study offer complementary information. RSA is a useful tool to enable the comparison of neural representations measured by different neuroimaging modalities (Kriegeskorte and Kievit, 2013). By creating similarity structures from EEG and fMRI activity patterns in the same format, it becomes possible to directly correlate the representational geometries emerging in neural space and time. However, the RSA-based data fusion is in itself blind to the informational content that is driving the match in representations (Schyns et al., 2020). Classification-based approaches on fMRI (or EEG) data, on the other hand, can pinpoint categorical representations that are explicitly built into the experimental design. However, they are restricted to very few categories (e.g., animacy, color), in turn limiting the conclusions that can be drawn about the certainly much richer and more multidimensional content of mental (memory) representations. In sum, data fusion and categorical decoding methods offer complementary insights into the content of reactivated memories, likely explaining why, in our study, some correlation peaks in the searchlight fusion maps during retrieval did not overlap with the sources of either perceptual or conceptual features. Richer study designs with more stimulus variations on a trial-by-trial basis (Schyns et al., 2020) are a promising avenue for revealing what type of content representations are contained in, and dominate, reactivated memories. An exciting recent development is the use of deep neural networks (DNNs) trained on image categorization and to compare the networks’ layer patterns with the representational patterns that emerge in the real brain (Cichy et al., 2019; E. J. Allen et al., 2022). Some recent work has employed this approach to shed light onto the nature of reactivated memory representations, for example, to investigate what level of feature reactivation predicts the vividness and distinctiveness of a memory (Bone et al., 2020). Others have used DNNs in comparison with intracranial EEG to reveal memory transformations over time and across different processing stages (Liu et al., 2020). Finally, different types of models, including word embedding models, have been used to uncover the temporal and content structure of recall narratives and how it compares with the original encoding (Heusser et al., 2021). In contrast to the relative black box of DNN layers, the latter models offer more transparency and interpretability of the dimensions contained in a memory and a route to understanding how the recall of naturalistic, continuous events unfolds in time.

In summary, our EEG–fMRI data fusion supports the idea of a retrieval process that at least partially progresses in reverse along the ventral visual stream with respect to encoding. Relating these findings to the fMRI decoding, which indicate a dominance of conceptual representations in later ventral regions and some perceptual representations in lower-level areas, it is likely to assume a conceptual-to-perceptual processing gradient during memory retrieval. Additionally, we show that the backward trajectory of memory reconstruction is not limited to ventral visual stream regions involved during encoding but also engages multisensory frontoparietal areas at distinct stages of retrieval processing, in line with the notion of a representational format transformation between perception and memory. The present findings demonstrate how the fusion of temporally and spatially resolved methods can further our understanding of memory retrieval as a staged feature reconstruction process, tracking how the reconstruction of a memory trace unfolds in time and space.

## Data Availability

The EEG data acquired by Linde-Domingo et al. (2019) can be found under https://doi.org/10.17605/OSF.IO/327EK. fMRI data can only partly be made publicly available to guarantee full anonymity in accordance with participants’ consent. Group-level data and nonidentifiable individual-level data can be found on the Open Science Framework with the identifier https://doi.org/10.17605/OSF.IO/2T7SN. Consent for the reuse of data does not include commercial research. The custom code used in this study is available on the Open Science Framework with the identifier https://doi.org/10.17605/OSF.IO/2T7SN. Additional code associated with the EEG data by Linde-Domingo et al. (2019) can be found under https://doi.org/10.17605/OSF.IO/327EK.

## Synthesis

Reviewing Editor: James Howard, Brandeis University Decisions are customarily a result of the Reviewing Editor and the peer reviewers coming together and discussing their recommendations until a consensus is reached. When revisions are invited, a fact-based synthesis statement explaining their decision and outlining what is needed to prepare a revision will be listed below. The following reviewer(s) agreed to reveal their identity: Bradley Buchsbaum.

This study investigates the neural representation of the content of episodic memory in different brain regions over time using representational-similarity-based analyses of EEG and fMRI results. More specifically, the authors sought to investigate whether visual object memories can be decomposed into perceptual and conceptual components, and whether these components are equally reinstated, and whether these components are reactivated with different temporal trajectories. This is done by examining how object-based representational similarity patterns (RSA) in fMRI data covary with time-point-by-timepoint RSA patterns in EEG. By correlating the two RSA matrices (fMRI <-> EEG) across time a time course can be revealed. By examining the cumulative sum of this trajectory, some inferences can be made about the emergence of feature representations across time and space. The main idea is to capture the forward vs backward progression assumed to occur during encoding and retrieval respectively.

The intention of this study was to collect EEG and fMRI data simultaneously but the acquired EEG data was not of sufficiently good quality for analysis. Thus, the authors re-used data from a previously published EEG study to perform an analysis with an fMRI dataset from a new participant sample. The approach of using representational similarity analyses to combine very different types of data has been tried before. Nevertheless, this is still a new and innovative approach and the study may be of interest to researchers who use EEG, MEG and fMRI - not just those in the field of episodic memory. The authors faced challenges in carrying out the study, but these were explicitly identified by the authors, such as the "Analyses that did not work" section.

1. We acknowledge that the in-scanner EEG data was "noisy" and to some extent the analyses "didn't work" because simultaneous fMRI-EEG data collection is notoriously difficult. However, a more detailed description is needed regarding precisely what the authors think may have contributed to problems with the data collection and analyses. For example, the data could have been corrupted by artifacts that could not be filtered out, etc.

2. The "cumulative sum" approach to the data analysis captures the accumulation of values during periods where no correlation is occurring, and therefore accumulates noise. Please provide an explanation as to why this approach is better than other potential approaches, such as identifying peaks, etc. Is this method more reliable?

3. The sequential propagation idea makes sense when there is a reliable peak correlation for each ROI in both encoding and retrieval. However here, for encoding, there is only seemingly a reliable correlation for V1/V2 and hIT (Figure 4A). Please explain why this is not also the case for Temporal pole and MTL.

4. The test for sequential propagation is plotted in Figure 4, but the cumulative sums are not plotted. It would also be helpful to see the cumulative sums of each ROI to evaluate whether they are interpretable, since the slope of the sums at each time-point is a more complex derived measure that has an intercept term. This is also because in Figure 4C, the time-point correlations show little evidence of a progression. The correlations swing between negative and positive values and are bounded by t=-3 and t=3, which is not that large considering the number of comparisons.

5. Testing the propagation idea with linear regression on ordinal values is could be sensitive to detecting gradient driven entirely by, say, the first ROI in the sequence. A Spearman correlation would be less sensitive to this effect, but one would also want to explicitly check the data by visualizing these lines and the datapoint that they are fitted to. Probably a quadratic fit would be significant, if the line were nonlinear.

6. Normalizing the correlation time-courses to sum to 1 is a kind of correction for differences in SNR, but it also would seem to boost correlation values for "noisy" regions. In this case, what is the meaning of the cumulative sum? It would be a random process. We think it is important to establish that each ROI has a reliable association both within fMRI and for the EEG-fMRI fusion. There is weak evidence of this for the retrieval condition (we see some stars on Figure 4C/D, but hard-pressed to see that these are significant after any sort of correction).

7. Time-locking analysis to subjects' impression of retrieval makes some sense, but depends on this report being accurate. Why not time-lock to cue onset? Has this been tried?

8. It should be stated in the Abstract that the fMRI and the EEG data from separate studies and separate cohorts of participants.

9. In the Introduction, the manuscript is framed to be around "reinstatement", the idea that perceptual and conceptual representations that are active during memory encoding (perception), are then re-activated during memory. As the authors state, there are many studies showing evidence for reinstatement, using encoding-retrieval-similarities (ERS) to index this. Therefore, the more novel aim of the study is to identify the time course of reinstatement effects in different brain regions, with the hypothesis that this will flow in an anterior-to-posterior direction. An alternative view is that the processes or representations active during recall are different from those active during encoding - memory many be "transformed" or "semanticized" versions of what was originally experienced. Of course, both of these processes might be in play during memory recall. The Abstract and the paper in general argue firmly that the results support the reinstatement hypothesis. However, the results are only weakly supportive of this position and there is equally clear, if not clearer, support for the view that processes during retrieval are different from those during encoding. In revising the manuscript, we urge the authors to discuss these concepts throughout the paper rather than just in a paragraph in the Discussion.

10. The best evidence for the authors' hypothesis that during retrieval there is a backward reactivation of cortical regions is the linear regression on the peak time points of reactivated brain regions (Figure 4f). This is only found for the ventral regions: MTL (not including hippocampus) > Temporal pole > HIT > V1V2. Is there any precedent for selecting these particular ROIs and arranging them in this order? (Have the same ROIs been used in similar previous studies?).

11. For clarity in Figure 4 it would help if panels e and f are clearly labelled as showing propagation within the ventral stream ROIs.

12. Apart from the linear regression result, the overall evidence for reinstatement seems rather weak. For example, no regions were found which showed direct evidence for reinstatement in the form of encoding-retrieval-similarity effects. Moreover, there are substantial differences between the regions engaged during encoding and retrieval (see Figure 5). The authors suggest that the searchlight fusion approach shown in Figure 5 (together with the ROI approach) show a "progression of the peak correlations from fronto-temporal to posterior sensory regions, generally in line with a backward propagation hierarchy". This claim is not well-supported by the data, and we suggest that this description is revised.

13. We agree with the authors summary of their findings later in the Discussion. They state that "Our findings support the previously described encoding-retrieval distinction" and there is discussion of "representational transformation" and "semanticization". However, it is confusing for the reader to understand what the findings of the study really show (i.e. whether they support "backward propagation" or "encoding-retrieval distinction" or both). It would be helpful if these two views are introduced earlier in the manuscript and the evidence for (and against) both views from the study results is summarized.

14. There are other points where the interpretation of the findings don't seem to match particularly well with the results themselves. For example, during encoding, the consistent finding from both the ROI and the searchlight fusion analyses is that representations are activated quickly in the early visual cortical regions and they remain active for the period analysed (around 1.5 seconds). While there are references to the feedforward sweep of information and the clear forward progression of correlation patterns, it is never mentioned that visual areas V1 and V2 represent the object information most robustly (compared to other regions) throughout encoding.

15. Another example is where the authors conclude that, "our study supports the idea of a conceptual-to-perceptual information processing gradient that is reversed with respect to encoding along ventral visual stream regions processing object identity." However, the RDMs are carried out at the object level and generalize across different picture formats - line drawing or color photo. Therefore, are the results not equally compatible with the view that (purely) conceptual information is represented within the ventral visual stream and activated either in a feed-forward or feed-back manner during encoding and retrieval respectively?

16. A more specific point about the analysis shown in Figure 5: the figure seems to show brain regions where the object representational "structure" from the fMRI data is similar to the (group level) object representational structure from the EEG data at particular timepoints(?). Is any form of correction for multiple comparisons carried out for this analysis? If the clusters are significant at p<0.05, and analyses are carried out at every time bin, then presumably there is a high chance of false positive errors?

17. In general, throughout the manuscript, much is made of the larger conceptual versus perceptual decoding effects using the LDA of the fMRI data. However, these comparisons seem only to be qualitative comparisons between the number of voxels that exceed threshold for significance in the different analyses. Was there any evidence for significantly greater decoding of conceptual versus perceptual classes in any region?

18. The method section leaves it unclear whether the data from the 11 remaining simultaneous fMRI-EEG recording was used at all - or whether only the EEG data from the previous publication was used.

## Author Response

Synthesis of Reviews:

Computational Neuroscience Model Code Accessibility Comments for Author (Required):

N/A Synthesis Statement for Author (Required):

This study investigates the neural representation of the content of episodic memory in different brain regions over time using representational-similarity-based analyses of EEG and fMRI results. More specifically, the authors sought to investigate whether visual object memories can be decomposed into perceptual and conceptual components, and whether these components are equally reinstated, and whether these components are reactivated with different temporal trajectories. This is done by examining how object-based representational similarity patterns (RSA) in fMRI data covary with time-point-by-timepoint RSA patterns in EEG. By correlating the two RSA matrices (fMRI <-> EEG) across time a time course can be revealed. By examining the cumulative sum of this trajectory, some inferences can be made about the emergence of feature representations across time and space. The main idea is to capture the forward vs backward progression assumed to occur during encoding and retrieval respectively.

The intention of this study was to collect EEG and fMRI data simultaneously but the acquired EEG data was not of sufficiently good quality for analysis. Thus, the authors re-used data from a previously published EEG study to perform an analysis with an fMRI dataset from a new participant sample. The approach of using representational similarity analyses to combine very different types of data has been tried before. Nevertheless, this is still a new and innovative approach and the study may be of interest to researchers who use EEG, MEG and fMRI - not just those in the field of episodic memory. The authors faced challenges in carrying out the study, but these were explicitly identified by the authors, such as the "Analyses that did not work" section.

1. We acknowledge that the in-scanner EEG data was "noisy" and to some extent the analyses "didn't work" because simultaneous fMRI-EEG data collection is notoriously difficult. However, a more detailed description is needed regarding precisely what the authors think may have contributed to problems with the data collection and analyses. For example, the data could have been corrupted by artifacts that could not be filtered out, etc. >> We indeed think that remaining scanning artifacts corrupted the EEG signal in the simultaneous fMRI-EEG data, as described by the reviewers. We added an explanation to our manuscript that takes into account previous papers on prominent artifacts in simultaneous EEG-fMRI (Fellner et al., 2016) and the possible effects on EEG decoding (Vidaurre et al., 2012), see section 'Analyses that did not work/data fusion with the in-scanner EEG' - lines 591-596.

2. The "cumulative sum" approach to the data analysis captures the accumulation of values during periods where no correlation is occurring, and therefore accumulates noise. Please provide an explanation as to why this approach is better than other potential approaches, such as identifying peaks, etc. Is this method more reliable? >> The cumulative sum approach should be seen here as complementing the whole-brain fusion. It is particularly well suited to show the direction of information accumulation in cascade-like processes where it can be assumed that the information goes through an ordered series of processing stages. In our case, it can be used to investigate the relationship of cumulative sums between ROIs within participants and hence test a hypothesized order of ROI peaks across subjects. We added a few lines of description in section ('Analyses/EEG-fMRI data fusion') - lines 513-517.

According to the rationale of the cumulative sum approach (Michelmann et al., 2019), correlations based purely on noise would lead to cumulative sums fluctuating unsystematically, closely to and around the diagonal and be similarly large. In such a case, a linear regression per subject would result in average slopes equal to zero (added this info in lines 534-535). Significant deviations from this zero slope, as shown in our analyses, can thus not be explained by noise accumulation. If the reviewers/editor consider this useful, we could contact the authors of the cumulative sum method for their simulations supporting this point, and potentially include them in the supplements, though this might go beyond the purpose of the present paper.

3. The sequential propagation idea makes sense when there is a reliable peak correlation for each ROI in both encoding and retrieval. However here, for encoding, there is only seemingly a reliable correlation for V1/V2 and hIT (Figure 4A). Please explain why this is not also the case for Temporal pole and MTL. >> We appreciate there are no significant correlation peaks in temporal pole and MTL, limiting the interpretation of the cumulative sums. We assume the main reason for this lack of correlation is that vision is dominated by early perceptual peaks, which are strongly time-locked and much easier to pick up with EEG than the later processes, making it more difficult to map later ROIs in the RSA fusion. The forward propagation found during vision (encoding) is thus likely driven to a large degree by V1/V2 and hIT. We added a note on this in the discussion (lines 1094-1097). That said, we selected the set of ROIs for this analysis a-priori based on the literature, and with the goal of keeping a consistent set of regions across the encoding and retrieval fusions.

4. The test for sequential propagation is plotted in Figure 4, but the cumulative sums are not plotted. It would also be helpful to see the cumulative sums of each ROI to evaluate whether they are interpretable, since the slope of the sums at each time-point is a more complex derived measure that has an intercept term. This is also because in Figure 4C, the time-point correlations show little evidence of a progression. The correlations swing between negative and positive values and are bounded by t=-3 and t=3, which is not that large considering the number of comparisons. >> We appreciate this concern of the reviewers and added plots of the cumulative sums in the supplementary data (Extended Data, Fig. 4 - 2a-b). It is important to note that these are averages across participants and are thus only partially meaningful. Our main statistical evaluation, the linear regression analysis, is designed to calculate slopes for cumulative sums between ROIs within participants for each time point separately, thus taking into account individual ROI differences, before testing the significance of slopes across participants. These linear regression analyses, although resulting in very small slopes, show a statistically significant deviation from slopes that are equal to zero across participants (as already shown in Fig. 4e-f).

Further, we have adjusted Fig. 4 by using a Spearman correlation (see Point 5), which resulted in a few additional clusters, and by highlighting additional corrections for multiple comparisons (see Point 6).

5. Testing the propagation idea with linear regression on ordinal values is could be sensitive to detecting gradient driven entirely by, say, the first ROI in the sequence. A Spearman correlation would be less sensitive to this effect, but one would also want to explicitly check the data by visualizing these lines and the datapoint that they are fitted to. Probably a quadratic fit would be significant, if the line were nonlinear. >> We thank the reviewers for this comment. While we now use Spearman correlation for the fusion analyses (see Fig. 4), the cumulative sum method relies on a linear fit and especially on the slope of the fitting line (while the actual fit is not considered central). That said, regarding the goodness of linear vs non-linear fit, as shown in our newly added Extended Data: figure 4-2, examples of the peak time points do suggest a good linear fit. We are open to also reporting results for different (e.g. quadratic) fits if the reviewers consider it relevant, and have particular suggestions for what fits to include based on literature. We would also like to add that the peaks of the ROI time courses of retrieval in Fig.4c do suggest a backward processing in the order: MTL>temporal pole> hIT&V1/V2, suggesting it is not one particular ROI that drives the gradient during retrieval, which is the focus of the present paper.

6. Normalizing the correlation time-courses to sum to 1 is a kind of correction for differences in SNR, but it also would seem to boost correlation values for "noisy" regions. In this case, what is the meaning of the cumulative sum? It would be a random process. We think it is important to establish that each ROI has a reliable association both within fMRI and for the EEG-fMRI fusion. There is weak evidence of this for the retrieval condition (we see some stars on Figure 4C/D, but hard-pressed to see that these are significant after any sort of correction). >>We understand the reviewers' concern and fully acknowledge the noisy nature of the retrieval data. The following changes were made to substantiate our claims that there is meaningful signal in these noisy EEG patterns, while remaining transparent about major issues with the EEG-fMRI fusion:

First, we added a description on a possible scenario based on pure noise, which would lead to slopes equal to zero (also find our explanation in point 2), which will hopefully give more insight on the rationale behind the method (line 534-535). Second, the normalization (suggested by Michelmann et al., 2019) is indeed mainly done to make ROIs with differing correlation strengths comparable, which we clarify in 'Analyses/EEG-fMRI data fusion' line 519-521. In the same paragraph (lines 513-517) and in the discussion (lines 1089-1094), we also emphasise now that this sequence analysis only complements the fusion results and is not meant to be interpreted as a standalone result. Information about how strong correlations are in a given regions can be gained from other analyses, not the cumulative sum approach. We have tried to be very transparent about the fact that the fusion correlations are not very high for some regions in the first place, discussed as major weakness of the paper in the discussion (lines 1100-1102). We also adjusted fig. 4 to show that the retrieval peaks that do survive cluster correction (some additional ones now with Spearman correlations, see point 5) will indeed not survive an additional correction for multiple comparison when conducted on top of the cluster correction (fig. 4); this is also addressed in the discussion (lines 1102-1108).

7. Time-locking analysis to subjects' impression of retrieval makes some sense, but depends on this report being accurate. Why not time-lock to cue onset? Has this been tried? >> The decision to time-lock to the subjective button press was taken based on extensive analyses of this EEG dataset before first reporting the results in Linde-Domingo et al. (2019). These analyses showed that meaningful classification peaks could be identified when aligning to the time point of subjective recall, but not to the onset of the cue. When choosing the former approach, the decoding peaks for perceptual and semantic information (and their order) meaningfully matched behavioural patterns, paralleling the reaction times when subjects classified perceptual and semantic features, respectively. We are thus confident that the timing of these subjective button presses is meaningful, and aligning the EEG timelines to the button presses reduces noise. Other work from our lab (e.g., an MEG study by Kerren et al., 2022, eLife) similarly suggest that time-locking to this subjective response improves classification, likely by reducing temporal variability in the EEG patterns across trials that aids the decoder. If the reviewers do not see major issues with this approach, we would therefore like to keep this consistent rationale also for the present set of results.

8. It should be stated in the Abstract that the fMRI and the EEG data from separate studies and separate cohorts of participants. >> We thank the reviewers for pointing this out and adapted the abstract accordingly (lines 8, 13-14).

9. In the Introduction, the manuscript is framed to be around "reinstatement", the idea that perceptual and conceptual representations that are active during memory encoding (perception), are then re-activated during memory. As the authors state, there are many studies showing evidence for reinstatement, using encoding-retrieval-similarities (ERS) to index this. Therefore, the more novel aim of the study is to identify the time course of reinstatement effects in different brain regions, with the hypothesis that this will flow in an anterior-to-posterior direction. An alternative view is that the processes or representations active during recall are different from those active during encoding - memory many be "transformed" or "semanticized" versions of what was originally experienced. Of course, both of these processes might be in play during memory recall. The Abstract and the paper in general argue firmly that the results support the reinstatement hypothesis. However, the results are only weakly supportive of this position and there is equally clear, if not clearer, support for the view that processes during retrieval are different from those during encoding. In revising the manuscript, we urge the authors to discuss these concepts throughout the paper rather than just in a paragraph in the Discussion. >> Again, we thank the reviewers for this helpful comment and agree that our results only show partial support for veridical reinstatement, and suggest an additional transformation in terms of a relay to parietal and frontal areas. We now describe the two alternative processes in the abstract and introduction (lines 5-8, 15-24, 69-78), and discuss throughout where our results support one or the other alternative (1018-1021, 1035-1038, 1054-1056, 1104-1108, 1130-1142, 1189-1198).

10. The best evidence for the authors' hypothesis that during retrieval there is a backward reactivation of cortical regions is the linear regression on the peak time points of reactivated brain regions (Figure 4f). This is only found for the ventral regions: MTL (not including hippocampus) > Temporal pole > HIT > V1V2. Is there any precedent for selecting these particular ROIs and arranging them in this order? (Have the same ROIs been used in similar previous studies?). >> This order was indeed based on previous literature and we have added the relevant references accordingly (lines 539-540).

11. For clarity in Figure 4 it would help if panels e and f are clearly labelled as showing propagation within the ventral stream ROIs. >>We adjusted the headings of panels e &f accordingly.

12. Apart from the linear regression result, the overall evidence for reinstatement seems rather weak. For example, no regions were found which showed direct evidence for reinstatement in the form of encoding-retrieval-similarity effects. Moreover, there are substantial differences between the regions engaged during encoding and retrieval (see Figure 5). The authors suggest that the searchlight fusion approach shown in Figure 5 (together with the ROI approach) show a "progression of the peak correlations from fronto-temporal to posterior sensory regions, generally in line with a backward propagation hierarchy". This claim is not well-supported by the data, and we suggest that this description is revised. >> We agree this particular statement was ambiguous and was meant to refer to a backwards propagation in terms of feature hierarchies, from high-level fronto-temporal to lower-level sensory regions. This sentence is now changed (lines 985-990). We would also like to clarify that the data fusion (fig. 5) is blind to the informational content of representations (lines 1161-1162) and on top of that, treats encoding and retrieval data as separate. Hence, when speaking about encoding-retrieval overlap, we mainly refer to the fMRI LDA results, as discussed in lines 1035-1038, 1054-1056. Although no voxels survived cross-classification from encoding to retrieval or vice versa (lines 803-806), separate encoding and retrieval classification suggests overlap insofar as overlapping brain regions represent perceptual and/or conceptual features during the encoding and retrieval phase.

We agree that many data points support a transformation view. We added another figure in the supplementary files to indicate the partial overlap between encoding and retrieval in fMRI decoding analyses (Extended data: fig. 2 -1, lines 798-802). As mentioned in response to point 9, we otherwise made an attempt throughout the manuscript to tone down the emphasis on 'reinstatement' (or reverse reinstatement) and adjusted our conclusions accordingly (e.g., lines 987-990, 1018-1021, 1189-1198).

13. We agree with the authors summary of their findings later in the Discussion. They state that "Our findings support the previously described encoding-retrieval distinction" and there is discussion of "representational transformation" and "semanticization". However, it is confusing for the reader to understand what the findings of the study really show (i.e. whether they support "backward propagation" or "encoding-retrieval distinction" or both). It would be helpful if these two views are introduced earlier in the manuscript and the evidence for (and against) both views from the study results is summarized. >> We agree with the reviewers that these opposing views were not introduced and sufficiently discussed in the previous version of the manuscript. As mentioned in response to point 12, our study shows partial re-involvement of regions that were also activated during encoding along the ventral visual stream during retrieval, following a backward gradient (also see Lifanov et al. 2021 &lines 1100-1108). We also emphasise that information is not only relayed back to ventral visual but also to parietal and frontal areas, which would be in line with a representational transformation and semanticization, the latter supported by the prominence of conceptual feature decoding during retrieval. We have tried to introduce the concepts in the introduction (line 69-78) and to implement a more concise summary of the results (line 1189-1198), which will hopefully make it easier for the reader to understand where the results support backward reinstatement vs transformation.

14. There are other points where the interpretation of the findings don't seem to match particularly well with the results themselves. For example, during encoding, the consistent finding from both the ROI and the searchlight fusion analyses is that representations are activated quickly in the early visual cortical regions and they remain active for the period analysed (around 1.5 seconds). While there are references to the feedforward sweep of information and the clear forward progression of correlation patterns, it is never mentioned that visual areas V1 and V2 represent the object information most robustly (compared to other regions) throughout encoding. >> We thank the reviewers for this comment and explicitly mention the temporally extended EVC contributions in the results/discussion now (lines 897, 1083-1086). We additionally would like to point to the whole-brain fusion which does show a forward sweep in terms of order/onset of the correlations.

15. Another example is where the authors conclude that, "our study supports the idea of a conceptual-to-perceptual information processing gradient that is reversed with respect to encoding along ventral visual stream regions processing object identity." However, the RDMs are carried out at the object level and generalize across different picture formats - line drawing or color photo. Therefore, are the results not equally compatible with the view that (purely) conceptual information is represented within the ventral visual stream and activated either in a feed-forward or feed-back manner during encoding and retrieval respectively? >> Indeed, as mentioned in point 12 (&lines 1161-1162), we can only make explicit points about perceptual and conceptual features in the decoding analyses of the fMRI data, while the fusion does not provide any information on which features contribute to the correlations. We adapted our conclusions to make clear that based on the fMRI data alone, we find conceptual dominance but also significant decoding of perceptual format during retrieval, suggesting it is not only conceptual information that is reactivated from memory. The fusion, on the other hand, suggests a backwards propagating stream during retrieval, involving regions already seen at encoding plus additional frontal/parietal areas (lines 1189-1198). A note on how perceptual features not under our experimental control might contribute to perceptual classification can also be found in lines 738-741).

16. A more specific point about the analysis shown in Figure 5: the figure seems to show brain regions where the object representational "structure" from the fMRI data is similar to the (group level) object representational structure from the EEG data at particular timepoints(?). Is any form of correction for multiple comparisons carried out for this analysis? If the clusters are significant at p<0.05, and analyses are carried out at every time bin, then presumably there is a high chance of false positive errors? >> Indeed, while these results are based on a stringent spatial cluster correction, there is no additional correction for multiple comparison over time. As mentioned before, we are aware this is a major weakness of the study and now make this more transparent in our discussion (lines 1101-1104).

17. In general, throughout the manuscript, much is made of the larger conceptual versus perceptual decoding effects using the LDA of the fMRI data. However, these comparisons seem only to be qualitative comparisons between the number of voxels that exceed threshold for significance in the different analyses. Was there any evidence for significantly greater decoding of conceptual versus perceptual classes in any region? >> We thank the reviewers for this comment and now added a statistical contrast between conceptual and perceptual searchlights, showing that there are higher accuracies for some regions classifying conceptual > perceptual categories (p < .001 uncorrected), however, there were none classifying perceptual > conceptual categories during retrieval (New extended Data:Table 2 - 6b, lines 788-794).

18. The method section leaves it unclear whether the data from the 11 remaining simultaneous fMRI-EEG recording was used at all - or whether only the EEG data from the previous publication was used. >> We thank the reviewers for noticing this and clarified accordingly that no EEG was used from the simultaneous recordings (lines 128).
