# Supplementary figures and images for: Reconstructing Spatiotemporal Trajectories of Visual Object Memories in the Human Brain
Source: eNeuro. 2024 Sep 26;11(9):ENEURO.0091-24.2024. doi: 10.1523/ENEURO.0091-24.2024 (PMC11439564; doi:10.1523/ENEURO.0091-24.2024)

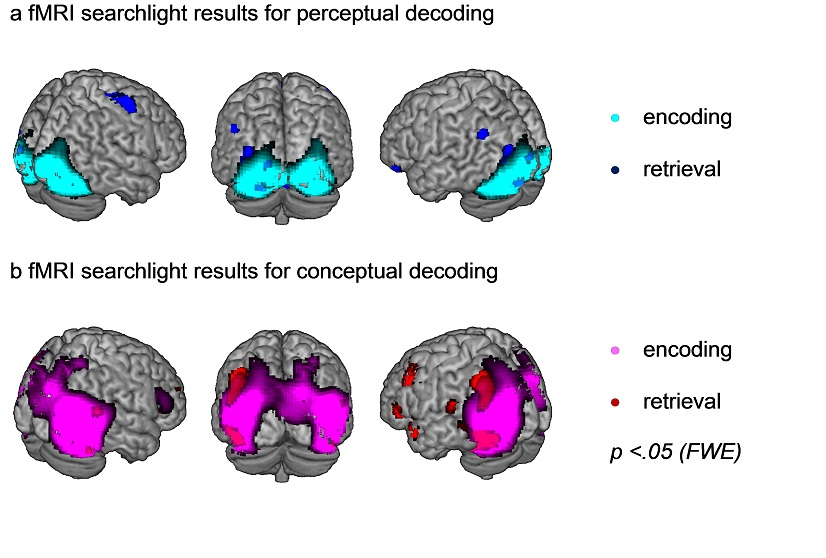

Supplement: Figure 2-1 — Searchlight LDA results illustrating encoding-retrieval overlap. Second-level t-contrasts show a) perceptual and b) conceptual accuracies significantly higher than the 50% chance level when classifying from the encoding (cyan/magenta) and retrieval (dark blue/red) data. All contrasts are thresholded at p < .05 (FWE-corrected). N = 31 independent subjects. Figure made using MRIcron (https://www.nitrc.org/projects/mricron, www.mricro.com, Rorden & Brett, 2000) and a colin 27 average brain template (http://www.bic.mni.mcgill.ca/ServicesAtlases/Colin27, Holmes et al., 1998; Copyright (C) 1993–2009 Louis Collins, McConnell Brain Imaging Centre, Montreal Neurological Institute, McGill University). Download Figure 2-1, TIF file. [file eneuro-11-ENEURO.0091-24.2024-s003.tif]

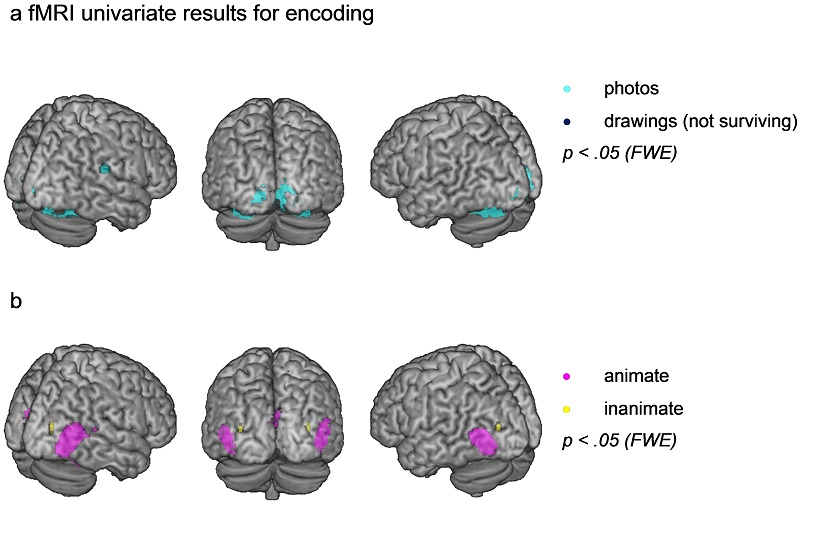

Supplement: Figure 2-2 — Univariate results. Second-level t-contrasts for encoding. a) Cyan: Photograph > drawing, dark blue: drawing > photograph. b) Magenta: animate > inanimate, yellow: inanimate> animate. All contrasts are thresholded at t(30) = 4.56, p < .05 (FWE). N = 31 independent subjects. During retrieval, no cortical voxels survived family-wise error correction (p < .05 FWE) when contrasting perceptual or conceptual categories. Figure made using MRIcron (https://www.nitrc.org/projects/mricron, www.mricro.com, Rorden & Brett, 2000) and a colin 27 average brain template (http://www.bic.mni.mcgill.ca/ServicesAtlases/Colin27, Holmes et al., 1998; Copyright (C) 1993–2009 Louis Collins, McConnell Brain Imaging Centre, Montreal Neurological Institute, McGill University). Download Figure 2-2, TIF file. [file eneuro-11-ENEURO.0091-24.2024-s005.tif]

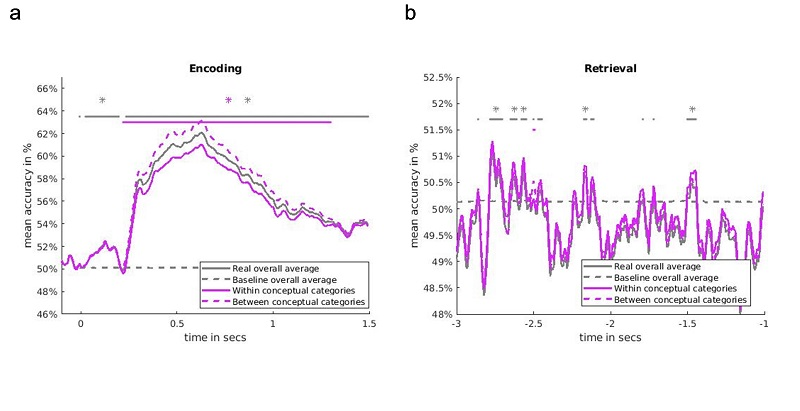

Supplement: Figure 4-1 — Average accuracy of EEG-based classification of object identity over time (grey), and average classification accuracy within- versus between conceptual classes (magenta) during a) encoding and b) retrieval. At encoding, time point 0 s marks the object onset. At retrieval, time point 0 s marks the button press, but is not included in figure as it does not lie within the time window of interest (see methods). Solid grey line represents average accuracy across the entire RDM, dashed grey line represents baseline average, and grey markers indicate significant overall classification accuracy against chance through random label permutation, compared by independent-sample t-tests. Solid magenta line represents the average accuracy within conceptual categories, dashed magenta line represents average accuracy between conceptual categories, and magenta markers indicate significant between- versus within conceptual classes with correct labels, compared by paired-sample t-tests. Significant decoding accuracy is indicated by points (p < .01 unc.) and asterisk (p < .05 cluster). See Methods for details on cluster correction. N = 24 independent subjects. Download Figure 4-1, TIF file. [file eneuro-11-ENEURO.0091-24.2024-s006.tif]

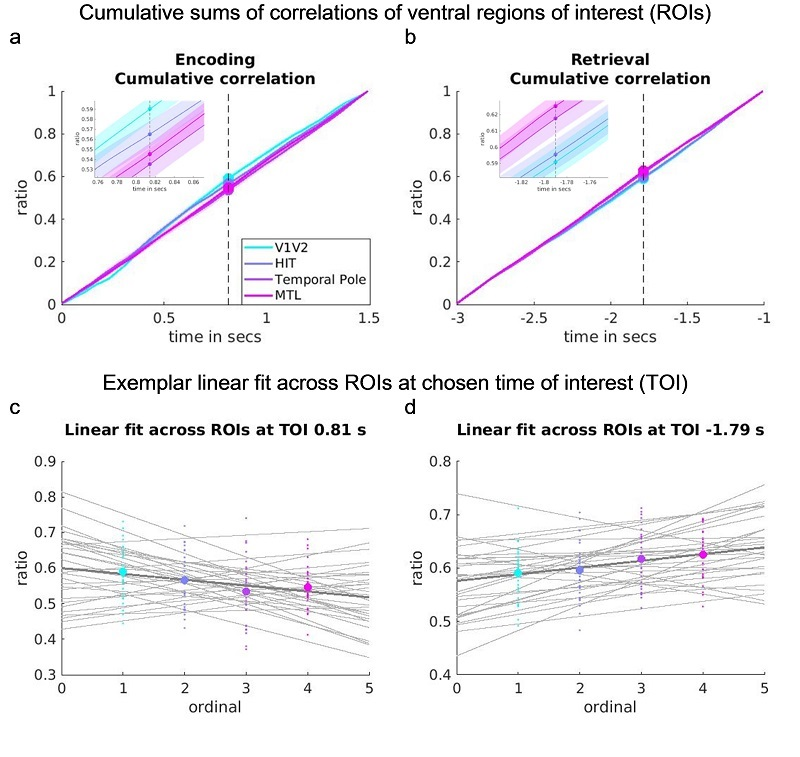

Supplement: Figure 4-2 — Region of interest (ROI) fusion: linear fit. A-b) Average cumulative sums of correlations and standard error (in zoomed in version) across subjects within a) encoding and b) retrieval. Dashed vertical line indicates the time point of minimum beta value for encoding (0.81 s) and maximum beta value for retrieval (-1.79 s). c-d) A linear fit of the cumulative sums of correlation time courses across ROIs within the ventral visual stream. A linear fit was made for an exemplar TOI (time of interest), 0.81 s for encoding c) and -1.79 s for retrieval d). Plots showing average ratio (thick dots) and average slope (thick line), as well as ratio and slopes within participants (small dots, thin lines). ROIs are color-coded as in legend. A negative slope suggests that earlier ROIs along the ventral visual stream have a higher cumulative sum than later ROIs, indicative of a forward stream. According to the same logic, a positive slope indicates a backward stream. Method adapted from Michelmann et al., 2019. In a-b), time point 0 s marks the object onset during encoding, and the subjective recall button press during retrieval. The latter is not included on time axis as it does not lie within the time window of interest (see Methods, also for details on cluster correction). Variance comes from n = 31 independent subjects. Download Figure 4-2, TIF file. [file eneuro-11-ENEURO.0091-24.2024-s007.tif]
