# Supplementary material for: Reconstructing Spatiotemporal Trajectories of Visual Object Memories in the Human Brain
Source: eNeuro. 2024 Sep 26;11(9):ENEURO.0091-24.2024. doi: 10.1523/ENEURO.0091-24.2024 (PMC11439564; doi:10.1523/ENEURO.0091-24.2024)
Supplement: Table 2-3 — fMRI searchlight results for encoding: perceptual features. Download Table 2-3, DOC file. [file eneuro-11-ENEURO.0091-24.2024-s008.doc]

| fMRI searchlight results for encoding: perceptual features  Statistics: p-values adjusted for search volume | | | | | | | | | | | | | |
| --- | --- | --- | --- | --- | --- | --- | --- | --- | --- | --- | --- | --- | --- |
| set-level | | cluster-level | | | | peak-level | | | | | x | y | z |
| p | c | p(FWE-corr) | q(FDR-corr) | k_E_ | p(unc) | p(FWE-corr) | q(FDR-corr) | T | equivZ | p(unc) | mm | mm | mm |
| 0.050 | 1 | 0.000 | 0.000 | 4298 | 0.000 | 0.000 | 0.000 | 15.89 | Inf | 0.000 | -30 | -64 | -21 |
|  |  |  |  |  |  | 0.000 | 0.000 | 15.59 | Inf | 0.000 | 24 | -88 | -7 |
|  |  |  |  |  |  | 0.000 | 0.000 | 15.30 | Inf | 0.000 | 30 | -76 | -14 |
