# Supplementary material for: Reconstructing Spatiotemporal Trajectories of Visual Object Memories in the Human Brain
Source: eNeuro. 2024 Sep 26;11(9):ENEURO.0091-24.2024. doi: 10.1523/ENEURO.0091-24.2024 (PMC11439564; doi:10.1523/ENEURO.0091-24.2024)
Supplement: Table 2-4 — fMRI searchlight results for encoding: conceptual features. Download Table 2-4, DOC file. [file eneuro-11-ENEURO.0091-24.2024-s009.doc]

| fMRI searchlight results for encoding: conceptual features  Statistics: p-values adjusted for search volume | | | | | | | | | | | | | |
| --- | --- | --- | --- | --- | --- | --- | --- | --- | --- | --- | --- | --- | --- |
| set-level | | cluster-level | | | | peak-level | | | | | x | y | z |
| p | c | p(FWE-corr) | q(FDR-corr) | k_E_ | p(unc) | p(FWE-corr) | q(FDR-corr) | T | equivZ | p(unc) | mm | mm | mm |
| 0.000 | 3 | 0.000 | 0.000 | 7638 | 0.000 | 0.000 | 0.000 | 20.98 | Inf | 0.000 | 45 | -73 | 4 |
|  |  |  |  |  |  | 0.000 | 0.000 | 16.79 | Inf | 0.000 | -39 | -79 | 7 |
|  |  |  |  |  |  | 0.000 | 0.000 | 16.71 | Inf | 0.000 | 42 | -61 | -21 |
|  |  | 0.000 | 0.007 | 212 | 0.005 | 0.003 | 0.054 | 6.12 | 4.89 | 0.000 | 54 | 26 | 14 |
|  |  | 0.033 | 0.645 | 5 | 0.645 | 0.019 | 0.383 | 5.24 | 4.38 | 0.000 | -6 | 68 | 21 |
