# Supplementary material for: Reconstructing Spatiotemporal Trajectories of Visual Object Memories in the Human Brain
Source: eNeuro. 2024 Sep 26;11(9):ENEURO.0091-24.2024. doi: 10.1523/ENEURO.0091-24.2024 (PMC11439564; doi:10.1523/ENEURO.0091-24.2024)
Supplement: Table 2-5 — fMRI searchlight results for retrieval: perceptual features. Download Table 2-5, DOC file. [file eneuro-11-ENEURO.0091-24.2024-s010.doc]

| fMRI searchlight results for retrieval: perceptual features  Statistics: p-values adjusted for search volume | | | | | | | | | | | | | |
| --- | --- | --- | --- | --- | --- | --- | --- | --- | --- | --- | --- | --- | --- |
| set-level | | cluster-level | | | | peak-level | | | | | x | y | z |
| p | c | p(FWE-corr) | q(FDR-corr) | k_E_ | p(unc) | p(FWE-corr) | q(FDR-corr) | T | equivZ | p(unc) | mm | mm | mm |
| 0.000 | 21 | 0.000 | 0.034 | 301 | 0.002 | 0.000 | 0.075 | 7.04 | 5.37 | 0.000 | 27 | -19 | 60 |
|  |  | 0.005 | 0.296 | 65 | 0.099 | 0.004 | 0.593 | 5.86 | 4.75 | 0.000 | 24 | 26 | 14 |
|  |  | 0.002 | 0.276 | 103 | 0.043 | 0.006 | 0.593 | 5.74 | 4.68 | 0.000 | 6 | 8 | 60 |
|  |  | 0.000 | 0.044 | 237 | 0.004 | 0.006 | 0.593 | 5.71 | 4.66 | 0.000 | 15 | -43 | 28 |
|  |  |  |  |  |  | 0.011 | 0.610 | 5.47 | 4.52 | 0.000 | 24 | -52 | 42 |
|  |  | 0.005 | 0.296 | 67 | 0.094 | 0.007 | 0.593 | 5.68 | 4.64 | 0.000 | -9 | -28 | 0 |
|  |  | 0.006 | 0.323 | 56 | 0.123 | 0.011 | 0.610 | 5.48 | 4.52 | 0.000 | -21 | -91 | -18 |
|  |  | 0.003 | 0.276 | 93 | 0.053 | 0.012 | 0.610 | 5.44 | 4.50 | 0.000 | -45 | -61 | 32 |
|  |  |  |  |  |  | 0.027 | 0.745 | 5.08 | 4.28 | 0.000 | -39 | -55 | 14 |
|  |  | 0.008 | 0.362 | 47 | 0.155 | 0.012 | 0.610 | 5.44 | 4.50 | 0.000 | 6 | -70 | -18 |
|  |  | 0.003 | 0.276 | 83 | 0.066 | 0.014 | 0.610 | 5.37 | 4.46 | 0.000 | -9 | -28 | 28 |
|  |  |  |  |  |  | 0.034 | 0.783 | 4.97 | 4.21 | 0.000 | -6 | -46 | 18 |
|  |  | 0.019 | 0.561 | 18 | 0.374 | 0.015 | 0.610 | 5.32 | 4.43 | 0.000 | -3 | 8 | 7 |
|  |  | 0.012 | 0.494 | 31 | 0.243 | 0.016 | 0.610 | 5.30 | 4.42 | 0.000 | 33 | 32 | 0 |
|  |  | 0.013 | 0.494 | 29 | 0.259 | 0.020 | 0.702 | 5.20 | 4.35 | 0.000 | -36 | -88 | 18 |
|  |  |  |  |  |  | 0.035 | 0.783 | 4.96 | 4.20 | 0.000 | -42 | -85 | 7 |
|  |  | 0.029 | 0.697 | 8 | 0.564 | 0.021 | 0.702 | 5.18 | 4.35 | 0.000 | -15 | -100 | 10 |
|  |  | 0.021 | 0.586 | 15 | 0.419 | 0.023 | 0.714 | 5.14 | 4.32 | 0.000 | -51 | -46 | -7 |
|  |  | 0.017 | 0.561 | 22 | 0.325 | 0.024 | 0.714 | 5.12 | 4.31 | 0.000 | -15 | -55 | 32 |
|  |  | 0.029 | 0.697 | 8 | 0.564 | 0.030 | 0.783 | 5.02 | 4.25 | 0.000 | 36 | -40 | -4 |
|  |  | 0.019 | 0.561 | 18 | 0.374 | 0.031 | 0.783 | 5.01 | 4.24 | 0.000 | -48 | 38 | -14 |
|  |  | 0.037 | 0.821 | 3 | 0.742 | 0.033 | 0.783 | 4.99 | 4.22 | 0.000 | -24 | -70 | -32 |
|  |  | 0.035 | 0.813 | 4 | 0.697 | 0.041 | 0.851 | 4.89 | 4.16 | 0.000 | 24 | -10 | -38 |
|  |  | 0.043 | 0.867 | 1 | 0.867 | 0.041 | 0.851 | 4.89 | 4.16 | 0.000 | -39 | 14 | -4 |
|  |  | 0.043 | 0.867 | 1 | 0.867 | 0.048 | 0.967 | 4.82 | 4.11 | 0.000 | 45 | -79 | 18 |
