# Supplementary material for: Reconstructing Spatiotemporal Trajectories of Visual Object Memories in the Human Brain
Source: eNeuro. 2024 Sep 26;11(9):ENEURO.0091-24.2024. doi: 10.1523/ENEURO.0091-24.2024 (PMC11439564; doi:10.1523/ENEURO.0091-24.2024)
Supplement: Table 2-6 — fMRI searchlight results for retrieval: a) conceptual features and b) conceptual features > perceptual features. Download Table 2-6, DOC file. [file eneuro-11-ENEURO.0091-24.2024-s011.doc]

| a) fMRI searchlight results for retrieval: conceptual features  Statistics: p-values adjusted for search volume | | | | | | | | | | | | | |
| --- | --- | --- | --- | --- | --- | --- | --- | --- | --- | --- | --- | --- | --- |
| set-level | | cluster-level | | | | peak-level | | | | | x | y | z |
| p | c | p(FWE-corr) | q(FDR-corr) | k_E_ | p(unc) | p(FWE-corr) | q(FDR-corr) | T | equivZ | p(unc) | mm | mm | mm |
| 0.000 | 12 | 0.000 | 0.000 | 1304 | 0.000 | 0.000 | 0.130 | 6.81 | 5.25 | 0.000 | -36 | 11 | 32 |
|  |  |  |  |  |  | 0.002 | 0.214 | 5.96 | 4.80 | 0.000 | -24 | 14 | -28 |
|  |  |  |  |  |  | 0.003 | 0.214 | 5.84 | 4.74 | 0.000 | 3 | 26 | -14 |
|  |  | 0.008 | 0.448 | 91 | 0.151 | 0.002 | 0.214 | 6.10 | 4.88 | 0.000 | -3 | -25 | -38 |
|  |  | 0.000 | 0.010 | 571 | 0.002 | 0.003 | 0.214 | 5.78 | 4.70 | 0.000 | -45 | -70 | -14 |
|  |  |  |  |  |  | 0.004 | 0.236 | 5.66 | 4.63 | 0.000 | -39 | -49 | -21 |
|  |  |  |  |  |  | 0.023 | 0.628 | 4.95 | 4.20 | 0.000 | -57 | -34 | 21 |
|  |  | 0.002 | 0.147 | 210 | 0.037 | 0.007 | 0.340 | 5.46 | 4.51 | 0.000 | -45 | -58 | 46 |
|  |  |  |  |  |  | 0.018 | 0.577 | 5.06 | 4.27 | 0.000 | -54 | -67 | 24 |
|  |  |  |  |  |  | 0.018 | 0.577 | 5.05 | 4.26 | 0.000 | -51 | -70 | 32 |
|  |  | 0.010 | 0.448 | 76 | 0.187 | 0.014 | 0.541 | 5.17 | 4.34 | 0.000 | 27 | -61 | 7 |
|  |  | 0.037 | 0.881 | 6 | 0.734 | 0.019 | 0.577 | 5.04 | 4.26 | 0.000 | 51 | -58 | -28 |
|  |  | 0.021 | 0.811 | 30 | 0.405 | 0.023 | 0.628 | 4.95 | 4.20 | 0.000 | 57 | -52 | 14 |
|  |  | 0.035 | 0.881 | 8 | 0.688 | 0.036 | 0.860 | 4.76 | 4.07 | 0.000 | -12 | 8 | 49 |
|  |  | 0.029 | 0.881 | 15 | 0.566 | 0.037 | 0.860 | 4.73 | 4.06 | 0.000 | -24 | 29 | 49 |
|  |  | 0.036 | 0.881 | 7 | 0.710 | 0.041 | 0.890 | 4.70 | 4.03 | 0.000 | 0 | -19 | -4 |
|  |  | 0.043 | 0.911 | 2 | 0.862 | 0.043 | 0.891 | 4.68 | 4.02 | 0.000 | 42 | 53 | 21 |
|  |  | 0.046 | 0.911 | 1 | 0.911 | 0.049 | 0.973 | 4.62 | 3.98 | 0.000 | 6 | 62 | 0 |
| b) fMRI searchlight results for retrieval: conceptual features > perceptual features  Statistics: p-values adjusted for search volume | | | | | | | | | | | | | |
| set-level | | cluster-level | | | | peak-level | | | | | x | y | z |
| p | c | p(FWE-corr) | q(FDR-corr) | k_E_ | p(unc) | p(FWE-corr) | q(FDR-corr) | T | equivZ | p(unc) | mm | mm | mm |
| 0.480 | 4 | 0.600 | 0.956 | 332 | 0.256 | 0.480 | 0.594 | 3.46 | 3.15 | 0.001 | -30 | 35 | 49 |
|  |  |  |  |  |  | 0.515 | 0.594 | 3.41 | 3.11 | 0.001 | -36 | 29 | 49 |
|  |  |  |  |  |  | 0.602 | 0.594 | 3.28 | 3.01 | 0.001 | -36 | 35 | 42 |
|  |  | 0.959 | 0.956 | 8 | 0.893 | 0.915 | 0.965 | 2.71 | 2.54 | 0.005 | -54 | 11 | -21 |
|  |  | 0.967 | 0.956 | 2 | 0.956 | 0.962 | 0.979 | 2.52 | 2.39 | 0.009 | -48 | 23 | -38 |
|  |  | 0.967 | 0.956 | 2 | 0.956 | 0.970 | 0.979 | 2.47 | 2.34 | 0.010 | -15 | -70 | 52 |
