# Supplementary material for: Reconstructing Spatiotemporal Trajectories of Visual Object Memories in the Human Brain
Source: eNeuro. 2024 Sep 26;11(9):ENEURO.0091-24.2024. doi: 10.1523/ENEURO.0091-24.2024 (PMC11439564; doi:10.1523/ENEURO.0091-24.2024)
Supplement: Table 2-7 — fMRI univariate results for encoding: photos > drawings. Download Table 2-7, DOC file. [file eneuro-11-ENEURO.0091-24.2024-s001.doc]

| fMRI univariate results for encoding: photos > drawings  Statistics: p-values adjusted for search volume | | | | | | | | | | | | | |
| --- | --- | --- | --- | --- | --- | --- | --- | --- | --- | --- | --- | --- | --- |
| set-level | | cluster-level | | | | peak-level | | | | | x | y | z |
| p | c | p(FWE-corr) | q(FDR-corr) | k_E_ | p(unc) | p(FWE-corr) | q(FDR-corr) | T | equivZ | p(unc) | mm | mm | mm |
| 0.000 | 7 | 0.000 | 0.000 | 1535 | 0.000 | 0.000 | 0.000 | 14.69 | Inf | 0.000 | 33 | -70 | -16 |
|  |  |  |  |  |  | 0.000 | 0.000 | 11.97 | Inf | 0.000 | -30 | -58 | -19 |
|  |  |  |  |  |  | 0.000 | 0.000 | 11.69 | Inf | 0.000 | -12 | -97 | -4 |
|  |  | 0.000 | 0.018 | 28 | 0.005 | 0.001 | 0.028 | 5.50 | 5.41 | 0.000 | 12 | -25 | 41 |
|  |  | 0.002 | 0.072 | 15 | 0.031 | 0.002 | 0.067 | 5.29 | 5.21 | 0.000 | 63 | -34 | 23 |
|  |  | 0.010 | 0.263 | 5 | 0.188 | 0.014 | 0.377 | 4.89 | 4.82 | 0.000 | -36 | -13 | 32 |
|  |  | 0.010 | 0.263 | 5 | 0.188 | 0.031 | 0.669 | 4.69 | 4.63 | 0.000 | -54 | -43 | 26 |
|  |  | 0.020 | 0.471 | 2 | 0.403 | 0.032 | 0.669 | 4.68 | 4.62 | 0.000 | -6 | -31 | 41 |
|  |  | 0.029 | 0.564 | 1 | 0.564 | 0.036 | 0.717 | 4.64 | 4.59 | 0.000 | 24 | -22 | -7 |
