# Supplementary material for: Reconstructing Spatiotemporal Trajectories of Visual Object Memories in the Human Brain
Source: eNeuro. 2024 Sep 26;11(9):ENEURO.0091-24.2024. doi: 10.1523/ENEURO.0091-24.2024 (PMC11439564; doi:10.1523/ENEURO.0091-24.2024)
Supplement: Table 2-8 — fMRI univariate results for encoding: animate > inanimate. Download Table 2-8, DOC file. [file eneuro-11-ENEURO.0091-24.2024-s002.doc]

| fMRI univariate results for encoding: animate > inanimate  Statistics: p-values adjusted for search volume | | | | | | | | | | | | | |
| --- | --- | --- | --- | --- | --- | --- | --- | --- | --- | --- | --- | --- | --- |
| set-level | | cluster-level | | | | peak-level | | | | | x | y | z |
| p | c | p(FWE-corr) | q(FDR-corr) | k_E_ | p(unc) | p(FWE-corr) | q(FDR-corr) | T | equivZ | p(unc) | mm | mm | mm |
| 0.000 | 5 | 0.000 | 0.000 | 409 | 0.000 | 0.000 | 0.000 | 14.25 | Inf | 0.000 | 51 | -70 | 5 |
|  |  |  |  |  |  | 0.000 | 0.000 | 10.17 | Inf | 0.000 | 42 | -49 | -22 |
|  |  |  |  |  |  | 0.001 | 0.040 | 5.40 | 5.31 | 0.000 | 54 | -43 | 14 |
|  |  | 0.000 | 0.000 | 170 | 0.000 | 0.000 | 0.000 | 11.59 | Inf | 0.000 | -48 | -76 | 5 |
|  |  | 0.004 | 0.105 | 9 | 0.084 | 0.000 | 0.000 | 6.73 | 6.56 | 0.000 | -39 | -46 | -22 |
|  |  | 0.000 | 0.000 | 145 | 0.000 | 0.000 | 0.003 | 5.97 | 5.86 | 0.000 | 9 | -76 | 8 |
|  |  |  |  |  |  | 0.003 | 0.083 | 5.22 | 5.14 | 0.000 | -6 | -88 | 20 |
|  |  |  |  |  |  | 0.009 | 0.204 | 4.99 | 4.92 | 0.000 | 6 | -79 | 29 |
|  |  | 0.006 | 0.123 | 7 | 0.123 | 0.010 | 0.213 | 4.96 | 4.89 | 0.000 | 9 | -52 | 50 |
