# Supplementary material for: Reconstructing Spatiotemporal Trajectories of Visual Object Memories in the Human Brain
Source: eNeuro. 2024 Sep 26;11(9):ENEURO.0091-24.2024. doi: 10.1523/ENEURO.0091-24.2024 (PMC11439564; doi:10.1523/ENEURO.0091-24.2024)
Supplement: Table 2-9 — fMRI univariate results for encoding: inanimate > animate. Download Table 2-9, DOC file. [file eneuro-11-ENEURO.0091-24.2024-s004.doc]

| fMRI univariate results for encoding: inanimate > animate  Statistics: p-values adjusted for search volume | | | | | | | | | | | | | |
| --- | --- | --- | --- | --- | --- | --- | --- | --- | --- | --- | --- | --- | --- |
| set-level | | cluster-level | | | | peak-level | | | | | x | y | z |
| p | c | p(FWE-corr) | q(FDR-corr) | k_E_ | p(unc) | p(FWE-corr) | q(FDR-corr) | T | equivZ | p(unc) | mm | mm | mm |
| 0.000 | 6 | 0.000 | 0.000 | 119 | 0.000 | 0.000 | 0.000 | 9.61 | Inf | 0.000 | 30 | -46 | -13 |
|  |  | 0.000 | 0.000 | 70 | 0.000 | 0.000 | 0.000 | 6.74 | 6.57 | 0.000 | -27 | -49 | -16 |
|  |  |  |  |  |  | 0.000 | 0.000 | 6.51 | 6.36 | 0.000 | -27 | -58 | -16 |
|  |  | 0.006 | 0.185 | 7 | 0.123 | 0.001 | 0.042 | 5.45 | 5.36 | 0.000 | -33 | -85 | 17 |
|  |  | 0.001 | 0.053 | 16 | 0.026 | 0.004 | 0.107 | 5.19 | 5.11 | 0.000 | 36 | -82 | 17 |
|  |  | 0.029 | 0.564 | 1 | 0.564 | 0.044 | 0.939 | 4.59 | 4.54 | 0.000 | -48 | -58 | -10 |
|  |  | 0.029 | 0.564 | 1 | 0.564 | 0.047 | 0.939 | 4.58 | 4.52 | 0.000 | -39 | -37 | 35 |
